# Exploiting the bile acid binding protein as transporter of a Cholic Acid/Mirin bioconjugate for potential applications in liver cancer therapy

**DOI:** 10.1038/s41598-024-73636-w

**Published:** 2024-09-28

**Authors:** Giusy Tassone, Samuele Maramai, Marco Paolino, Stefania Lamponi, Federica Poggialini, Elena Dreassi, Elena Petricci, Stefano Alcaro, Cecilia Pozzi, Isabella Romeo

**Affiliations:** 1 Department of Biotechnology, Chemistry and Pharmacy, University of Siena, Via Aldo Moro 2, 53100 Siena, Italy; 2 Department of Health Science, Università “Magna Graecia” di Catanzaro, Campus “S. Venuta”, Viale Europa, 88100 Catanzaro, Italy; 3 Net4Science Academic Spin-Off, Università “Magna Graecia” di Catanzaro, Campus “S. Venuta”, Viale Europa, 88100 Catanzaro, Italy; 4 Consorzio Interuniversitario Risonanze Magnetiche di Metallo Proteine (CIRMMP), Via Sacconi 6, 50019 Sesto Fiorentino (FI), Italy

**Keywords:** Drug conjugates, Bile acids, Carriers, BABP, X-ray crystallography, Molecular dynamic simulation, DNA damage, MRN complex, Anticancer, Cancer, Chemical biology, Medicinal chemistry, Organic chemistry, Structure determination, Molecular modelling, X-ray crystallography, Analytical chemistry

## Abstract

Bioconjugation is one of the most promising strategies to improve drug delivery, especially in cancer therapy. Biomolecules such as bile acids (BAs) have been intensively explored as carriers, due to their peculiar physicochemical properties and biocompatibility. BAs trafficking is regulated by intracellular lipid-binding proteins and their transport in the liver can be studied using chicken liver Bile Acid-Binding Proteins (cL-BABPs) as a reference model. Therefore, we conceived the idea of developing a BA-conjugate with Mirin, an exonuclease inhibitor of Mre11 endowed with different anticancer activities, to direct its transport to the liver. Following computational analysis of various BAs in complex with cL-BABP, we identified cholic acid (CA) as the most promising candidate as carrier, leading to the synthesis of a novel bioconjugate named CA-M11. As predicted by computational data and confirmed by X-ray crystallographic studies, CA-M11 was able to accommodate into the binding pocket of BABP. Hence, it can enter BAs trafficking in the hepatic compartment and here release Mirin. The effect of CA-M11, evaluated in combination with varying concentrations of Doxorubicin on HepG2 cell line, demonstrated a significant increase in cell mortality compared to the use of the cytotoxic drug or Mirin alone, thus highlighting chemo-sensitizing properties. The promising results regarding plasma stability for CA-M11 validate its potential as a valuable agent or adjuvant for hepatic cancer therapy.

**Supplementary Information:**

The online version contains supplementary material available at 10.1038/s41598-024-73636-w.

## Introduction

**Fig. 1:**
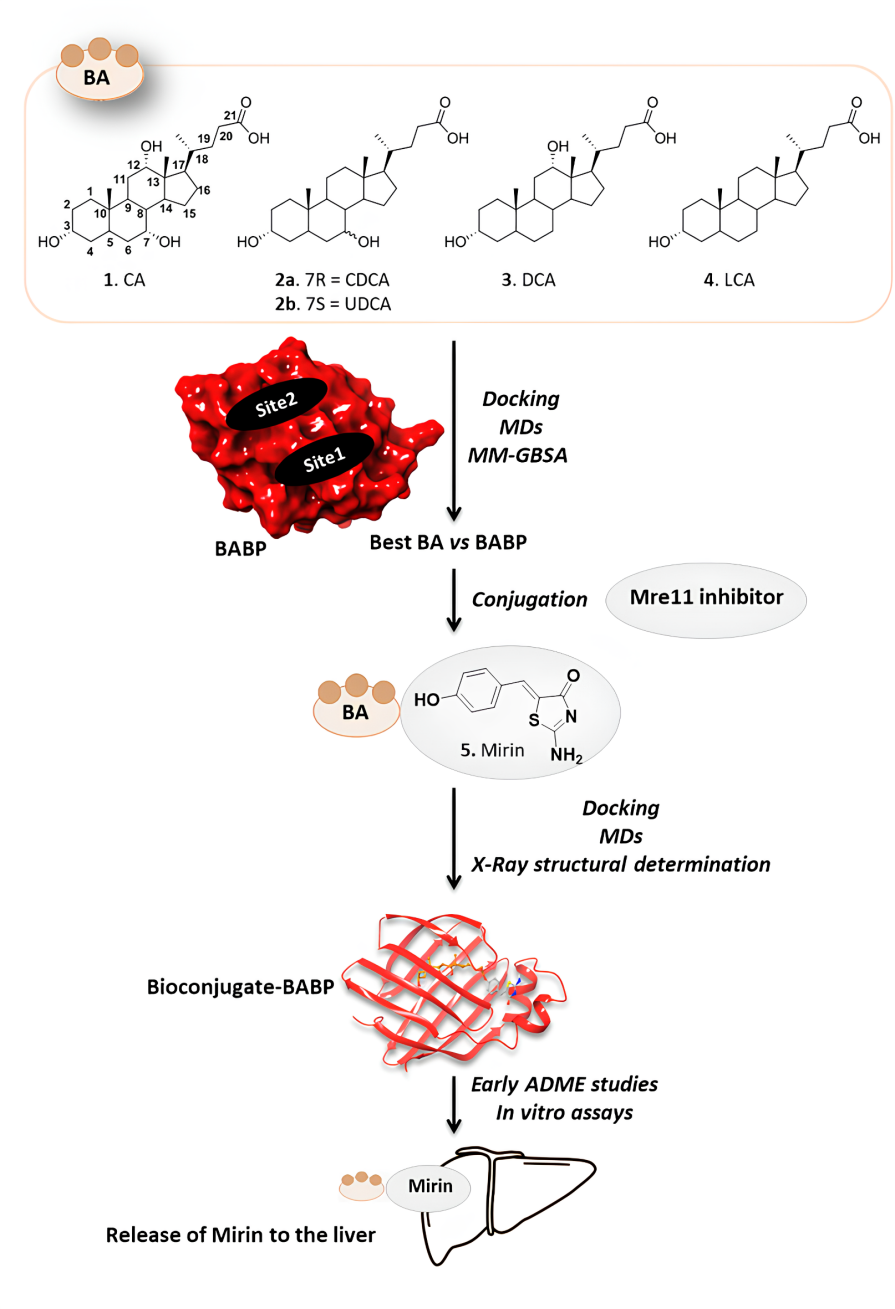
Workflow of the strategy to build a bioconjugate of Mirin with BAs for targeting the DNA repair protein Mre11 in the liver, exploiting iLBPs as transporters.

In the last decade, bioconjugation of small molecules to biocompatible carriers has proven to be an efficient approach for targeted delivery, controlled release, and distribution of drugs. This strategy encompasses the covalent linkage of drugs to biological macromolecules, thereby enhancing their solubility, stability, and pharmacokinetics while reducing side effects and minimizing toxicity^1^. Antibodies, aptamers, lipids, carbohydrates, and several other biologically active molecules have been exploited as efficient carriers to develop new molecular constructs for the therapy of different diseases. The selection of the carrier is of utmost importance since it can affect the drug release and absorption, thus impacting the efficacy and safety of the treatment. In this scenario, deep interest raised around bile acids (BAs) as drug carriers, in correlation to their biological significance and chemical properties^2,3^. BAs (**1–4**, Fig. 1) are amphipathic compounds produced from cholesterol by hepatocytes within the liver and actively transported and secreted into the bile canaliculi. The primary BAs, cholic acid (CA) and chenodeoxycholic acid (CDCA), can undergo further modification by gut flora, resulting in secondary BAs such as ursodeoxycholic acid (UDCA), deoxycholic acid (DCA) and lithocholic acid (LCA). Since the late 80’s, BAs have been used for building up bioconjugates with different applications. For instance, cytotoxic drugs such as chlorambucil^4^ or platinum-based anticarcinogens^5^ have been linked to different BAs. This strategy allowed to improve oral bioavailability and to direct them into the hepatobiliary compartment, contributing to overcome some limitations of the cytotoxic agents, such as toxicity and drug-resistance. Several other drugs with applications beyond cancer have been loaded on BAs, contributing to enhance intestinal absorption and liver targeting activity. However, to date, no BAs-based bioconjugate has entered the clinical development and additional studies related to their trafficking is still needed^6^. Interestingly, BAs trafficking is mediated by soluble intracellular lipid-binding proteins (iLBPs), which possess a ten-strand antiparallel β-cylinder and two α-helices defining an internal ligand-binding cavity suitable for interacting with BAs^7–10^. The iLBP family includes the cellular retinol-binding proteins (CRBPs), retinoic acid-binding proteins (CRABPs), and fatty acid-binding proteins (FABPs)^11–14^. Thus, the use of BAs as drug carriers paves the way for the bioconjugate to potentially interact with iLBPs and, therefore with FABPs. This interaction could facilitate its selective accumulation in certain body compartments, enabling the targeted release of the drug with enhanced efficacy, affinity, and selectivity toward its intended target. Inhibitors of the DNA-repair machinery at any level have a great potential as adjuvants in cancer treatment^15^, still finding an application in therapy limited to topoisomerase inhibitors^16^. At the cellular level, the maintenance of genomic stability relies on the DNA damage response (DDR), a multifaceted process involving various pathways and proteins able to halt cell cycle progression and to facilitate the repair or bypass of damages^17^. In this context, the Meiotic recombination 11 homolog 1 (Mre11)–DNA repair protein Rad50–Nijmegen breakage syndrome protein 1 (Nbs1) complex, better known as Mre11-Rad50-Nbs1 or MRN complex, plays a crucial role in sensing and signaling from DNA double-strand breaks^18^. Interfering with the MNR complex has been proposed as a strategy to induce cell death and treat cancer, albeit with limited application due to its ubiquitous expression in both healthy and tumor cells. However, Mre11 is often overexpressed in several tumors, impacting on topoisomerase II activity^19^, which has been linked to genomic instability in tumor cells^20^. Interestingly, the selective inhibition of Mre11 is reported to increase cancer sensitivity to radio- and chemotherapy^21,22^, being not compatible with cell survival^23,24^, and raised attention around this target for precision oncology therapies. Few molecules have been reported as Mre11 inhibitors and some of them have entered clinical trials for the treatment of different cancer diseases^22,24^. To date, no real applications for these compounds have been found, mainly because of their lack of selectivity or problems connected to pharmacokinetics and resistance mechanisms. However, the development of a bioconjugate charged with Mre11 inhibitors represents a concrete opportunity for the targeted delivery of these molecules that could find application in therapy even beyond cancer, being involved also in viral infections^25–27^. With the aim of developing a novel anticancer bioconjugate incorporating Mre11 inhibitors and BAs, we focused our attention on Mirin (**5**, Fig. 1) as the payload^28,29^. This compound is able to inhibit Mre11-associated MRN exonuclease activity and to abolish the G2/M checkpoint and homology-dependent repair in mammalian cells^28^. Treatment with Mirin alone has been shown to reduce tumor growth in neuroblastoma cells by increasing DNA damage^30^. Moreover, it compromised the clonogenic survival of colorectal cancer cells exposed to ionizing radiation, suggesting a synergistic effect for the two treatments^31^. However, before proceeding, we needed to identify the most suitable BA capable of serving as the carrier. As mentioned before, BAs could potentially provide a “sensor side” on the new bioconjugate to become a substrate of iLBPs, including FABPs. To test this hypothesis, we investigated on the interaction of five BAs with liver bile acid binding proteins (L-BABPs), a subset of cytosolic proteins found in various vertebrates (although not in mammals)^32,33^. L-BABPs, including the protein isolated from chicken liver (cL-BABP), have been thoroughly investigated in nanotechnology as suitable transporters of exogenous and physiological substances, owing to their high similarity with certain variants of mammalian FABPs^7,34,35^. Here is reported our strategy (Fig. 1) for the design and synthesis of a new bioconjugate, named CA-M11, along with the computational analysis performed on BAs and CA-M11 and the X-ray structural characterization of the complex with cL-BABP. Further analysis has been performed to assess the biological activity of CA-M11 and its preliminary in vitro pharmacokinetics.

## Results and discussion

The use of biomolecules to selectively shuttle cytotoxic compounds in specific body tissues represents a promising strategy to treat cancer^36^. In this context, BAs have emerged as optimal carriers for the selective delivery of drugs into the liver, offering potential for the development of novel bioconjugates for hepatic cancer treatment^3,37^. In fact, BAs exhibit strong drug binding capabilities and can be modified with different payloads, enhancing their ability to interact with cells and promoting drug accumulation^6^. Plasma stability is therefore essential during the process of transport in the hepatic compartment. In addition, the interaction of these bioconjugates with iLBPs, which are considered efficient transporters of drugs in the cytoplasm, offers the opportunity to develop new therapeutics or diagnostics to better elucidate the molecular mechanisms involved in BAs transport and BA-related disorders^38^. In this context, we aim for a novel CA-Mirin bioconjugate which is stable in plasma and can release Mirin upon transportation and trafficking in the liver. These last two processes are granted by the CA sensor-side of the molecule which is able to bind to iLBPs protein family. To study this interaction, our study focused on cL-BABP as a model protein, due to its higher similarity with the human iLBPs counterpart and the extensive characterization over the years^39^.

### Molecular docking and MDs of BAs into cL-BABP

The X-ray structure of the protein in complex with CA^35^ was used as the model to set-up our theoretical studies. The structure was preprocessed, and its energy was minimized to perform the molecular recognition of the other BAs, namely CDCA, DCA, LCA, and UDCA, by using Glide SP protocol. As determined by previous experimental data, the stoichiometry of ligand binding for BAs was of two cholates per cL-BABP molecule^39^. Therefore, we focused on both binding sites to determine which the most favored one. The accuracy of the docking procedure was validated by re-docking CA in both sites, assessed in terms of Root mean square deviation (RMSD). Overlapping the native poses with the best docked conformations revealed RMSD values of 0.42 and 0.26 Å, for site 1 and 2, respectively. As a result, CA showed the better theoretical binding affinity versus site 2 of the protein rather than site 1. Subsequently, the five mentioned BAs were docked at the putative cL-BABP binding site 2 to obtain insights into their potential binding conformations. After visual inspection of the best docked poses, it emerged that all BAs were well accommodated into the cL-BABP binding cavity, based on the docking score, expressed in kcal/mol (Table 1).

**Table 1:** Glide SP scores of the best-docked poses of BAs, such as CA, CDCA, DCA, LCA, and UDCA towards cL-BABP site 2.

Compound	Glide SP score (kcal/mol)
CA	− 12.18
CDCA	− 10.89
DCA	− 12.11
LCA	− 10.86
UDCA	− 9.53

A common feature observed amongst these BAs was the ability of the carboxylate moiety to interact with Lys77. Additionally, all compounds formed an H-bond between the hydroxyl 3 (atom numbering is displayed in Fig. 1) and the side chain of Gln101, except for UDCA.

**Fig. 2:**
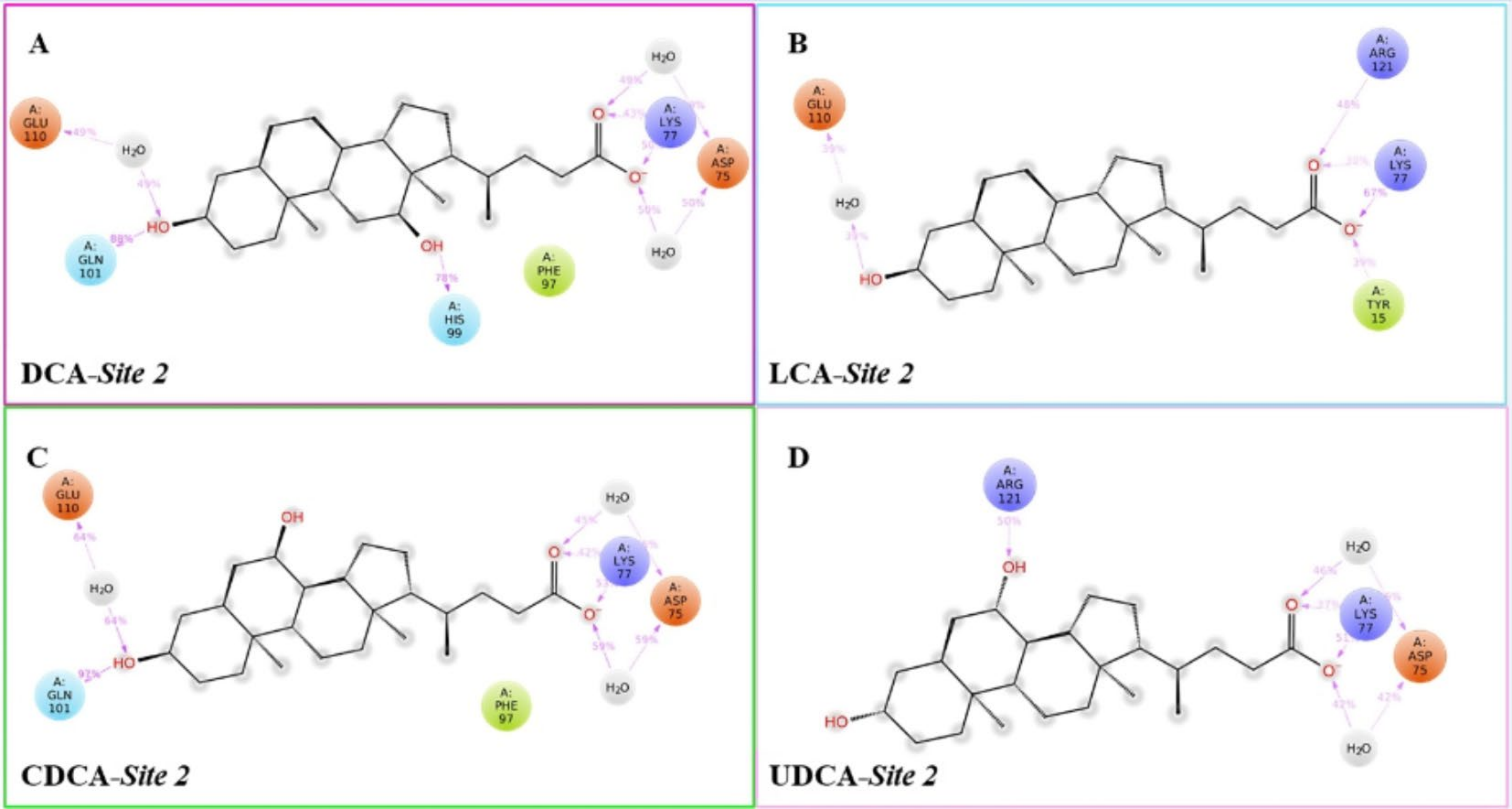
2D interaction diagrams of (**A**) DCA, (**B**) LCA, (**C**) CDCA, (**D**) UDCA in complex with cL-BABP site 2. The negative charged, the positive charged, the hydrophobic, the polar residues, and the water molecules are shown as red, blue, green, cyan, and grey spheres. The pink lines depict HB interactions followed by the related percentage of occurrence during the whole simulations.

**Fig. 3:**
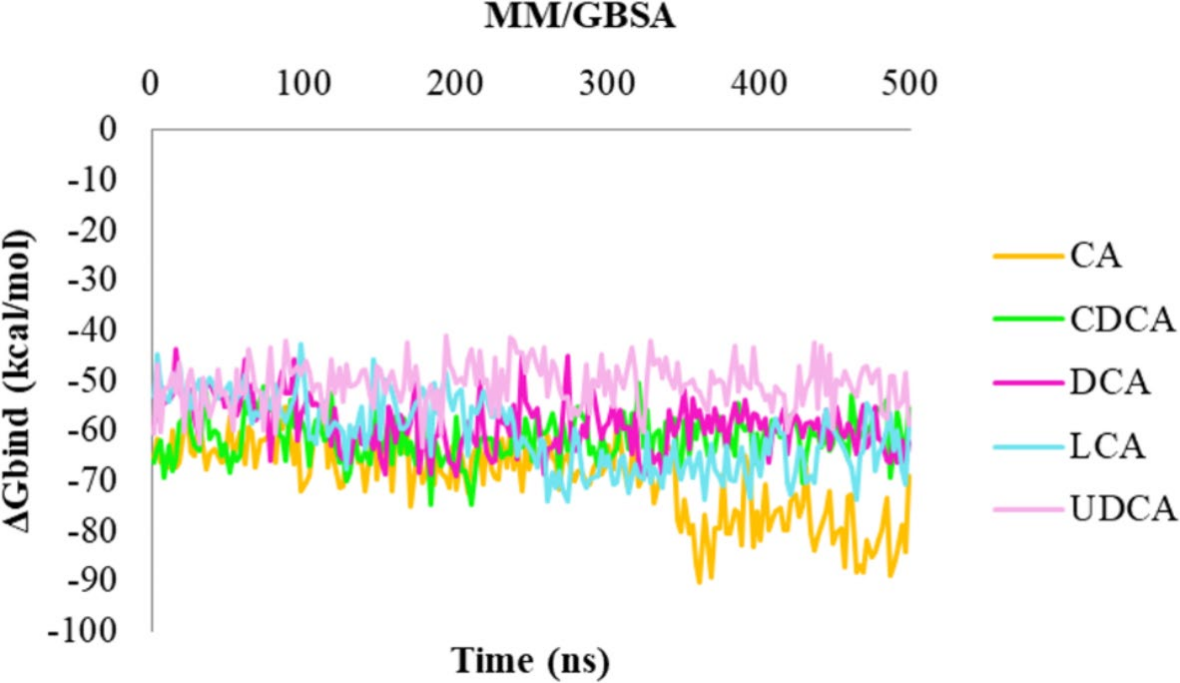
Plot of MM/GBSA trend for CA, CDCA, DCA, LCA, and UDCA in complex to cL-BABP, during 500 ns of MDs.

Meanwhile, the hydroxyl 12 of CA and DCA was anchored to His99 of cL-BABP (Figure S1, SI). At this point, the best-docked conformation for each investigated BAs into the protein was submitted to 500 ns of molecular dynamics simulations (MDs) to assess the quality of protein-ligand interactions (Fig. 2). In detail, by monitoring the whole MDs trajectories, it was observed that the H-bond between the hydroxyl 3 of CA, CDCA, DCA and Gln101 was maintained for 89%, 97%, and 88% of the MDs, respectively. This same hydroxyl group established a water-mediated interaction with Glu110 in all BAs, except for CA and UDCA. During 500 ns of MDs, it was also retained the interaction between hydroxyl 12 of CA (83%) and DCA (78%) with His99. Additional interactions were found between the carboxylate portion of all BAs and Asp75 (except for LCA), and Lys77 by means of a water-mediated interaction and an H-bond, respectively. Upon examining the dynamic behavior of the investigated BAs within cL-BABP, it was noted that Arg121 was directly bound to the hydroxyl 7 of UDCA and to the carboxylate group of LCA, but also mediated by a water-mediated interaction with CA. Finally, CA and LCA formed an H-bond and a water-mediated interaction with Tyr15 and Met74, respectively, only during 30% and 39% of MDs run (Fig. 2). MM-GBSA analysis upon the MDs trajectories enabled us to determine the binding free energy (ΔGbind) of each protein-ligand complex. 200 snapshots from the MD simulation were taken into account for each calculation. Among the BAs, the average effective free energy of binding was determined to be -69.61, -61.41, -58.80, -60.61, and − 50.72 kcal/mol for CA, CDCA, DCA, LCA, and UDCA in complex to cL-BABP, respectively (Fig. 3).

Our molecular docking and molecular dynamics simulations revealed insights into the binding mechanisms and stability of CDCA, DCA, LCA, UDCA, and CA with cL-BABP. Notably, residues Asp75, Lys77, His99, Gln101, and Glu110 emerged as key anchoring points for the investigated BAs throughout a substantial residence time of the MDs runs. The predicted binding mode of CA closely resembled the experimental structure of cL-BABP in complex with CA, as previously characterized^34,35^. It has become apparent that binding of BAs to cL-BABP requires the engagement of His99, Gln101, and Glu110 residues, located at site 2. MM/GBSA analysis highlighted significant differences in the binding free energy profiles of all examined complexes. Specifically, CA demonstrated a remarkable superior binding free energy profile compared to the other BAs, suggesting a stronger stabilizing effect and exhibited a longer residence time within the binding pocket of cL-BABP. In addition, CA is taken up by hepatocytes, making it suitable for shuttling drugs to the liver from the bloodstream^40^. This is essential in our design since it can allow the accumulation of our bioconjugate in the hepatic compartment while simultaneously enhancing its potential to serve as a substrate for FABPs Therefore, CA emerged as the optimal choice for our purposes.

### Crystal structures of cL-BABP in complex with BAs

**Fig. 4:**
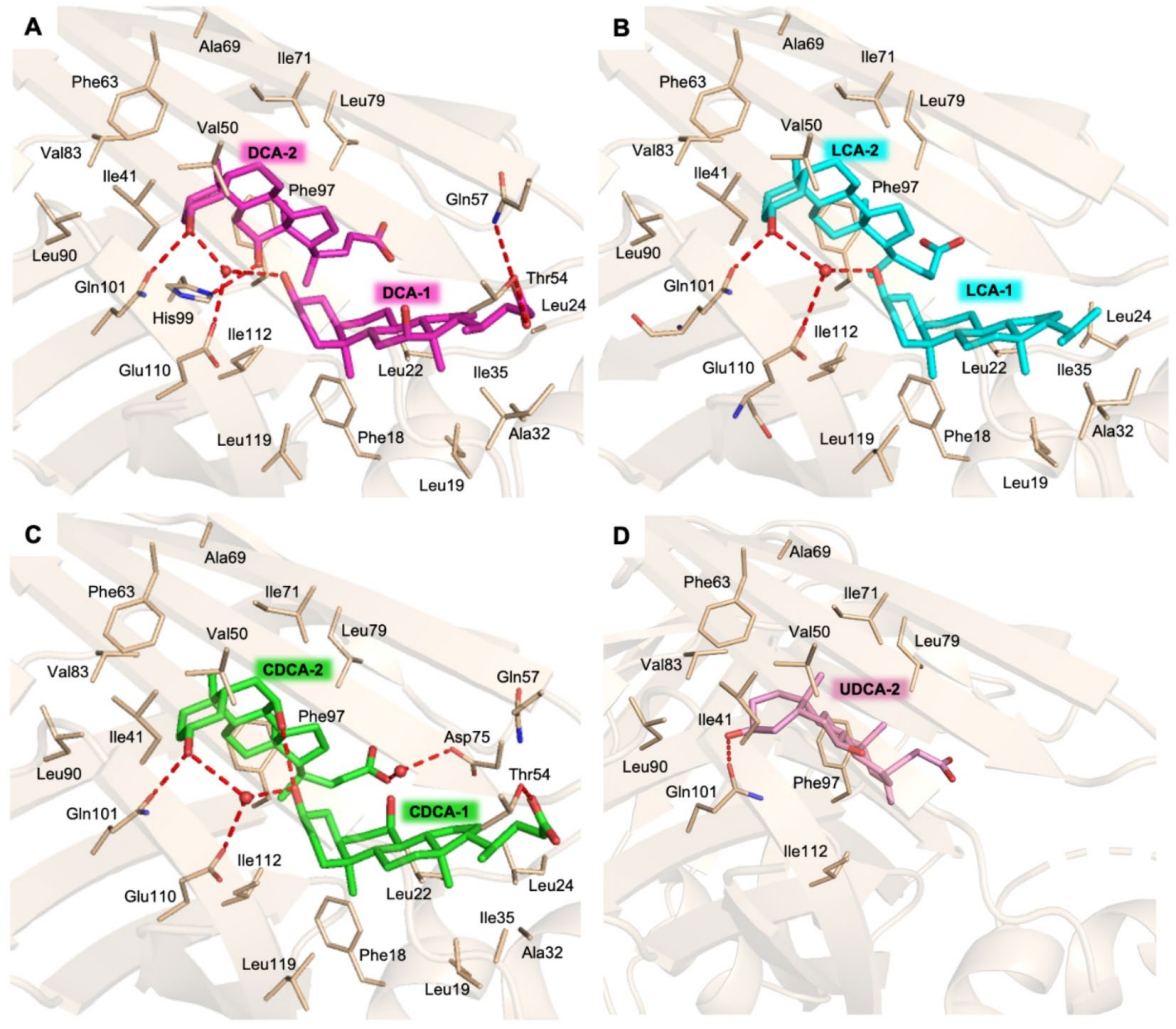
Crystal structure of recombinant cL-BABP (pale brown cartoon and carbons) in complex with BAs. The inner cavity of the protein is populated by two molecules, BA-1 and BA-2, except the complex with UDCA. Ligand-binding cavity of cL-BABP in complex with (**A**) DCA (in sticks, magenta carbons) (**B**) LCA (in sticks, cyan carbons) (**C**) CDCA (in sticks, green carbons), and (**D**) UDCA (in sticks, pink carbons). In all figures, water molecules are shown as red spheres and H-bonds as red dashed lines; oxygen atoms are colored red, nitrogen blue, and sulfur yellow.

DCA, LCA, UDCA, and CDCA were subjected to structural analysis to unveil their binding mode within the cL-BABP ligand-binding cavity. The structures of cL-BABP in complex with the four BAs were solved to resolutions ranging from 1.65 to 2.30 Å (Table S1). In all complexes, the crystal asymmetric unit is populated by two protein chains, labelled A and B. The overall fold consists of the canonical β-barrel with two five-stranded β-sheets and two α-helices connecting β1 and β2^35^. The ligand-binding cavity is large enough to accommodate two molecules of BAs (named BA-1 and BA-2) per protein chain in two distinct sites, defined as sites 1 and 2. This phenomenon is observed only in chain A of the structure in complex with DCA, LCA, and CDCA. However, in the BABP-UDCA structure, only one UDCA molecule occupies the ligand-binding site 2 in both chains. In all structures, the BAs are mainly stabilized by van der Waals interactions; nonetheless, their hydroxyl and carboxylate moieties form H-bonds with various inner cavity residues and with each other. DCA-1, LCA-1, and CDCA are accommodated in site 1, lined by residues belonging to the strands 1–6 and 10, as well as the two α-helices (Fig. 4, A-C). In this site, molecules entail extensive van der Waals interactions with residues such as Phe18, Leu19, Leu22, Leu24, Ala32, Ile35, Ala69, Ile112, and Leu119. The carboxylate moiety of DCA-1 and CDCA-1 forms H-bonds with Thr54 and Gln57. On the other hand, due to the lack of electron density, the carboxylic moiety of LCA-1 was not modelled. DCA-2, LCA-2, CDCA-2, and the sole molecule of UDCA (UDCA-2) occupy the second site, lined by residues of strands 2–9 (Fig. 4). All BAs in this site are mainly stabilized by van der Waal interactions involving residues like Ile41, Val50, Phe63, Ile71, Leu79, Val83, Leu90, and Phe97. The hydroxyl group 3 (atom numbering is displayed in Fig. 1) of all BAs entails an H-bond with Gln101. Furthermore, in DCA-2, LCA-2, and CDCA-2, this group forms a water-mediated interaction with Gln110 and with the same group of their counterpart in site 1 (Fig. 4, A-C). The carboxylate moiety of CDCA-2 forms a water mediated interaction with Asp75 and the CDCA-1 hydroxyl 7. The DCA-2 hydroxyl 12 engages an H-bond with His99. The CDCA-2 hydroxyl 7 entails an H-bond with CDCA-1 hydroxyl 3. Since the structure of the complex with UDCA contains only one acid molecule inside the ligand-binding cavity, the latter is rotated approximately 90° with respect to DCA, LCA, and CDCA. X-ray structural characterization of cL-BABP in complex with CDCA, UDCA, DCA, and LCA validated the in silico insights.

### Molecular recognition and MDs of conjugate CA-M11 into cL-BABP

**Fig. 5:**
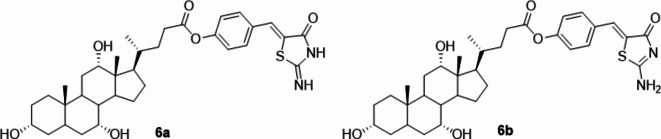
2D structure of iminothiazolidin-4-one tautomer **6a** and aminothiazolidin-4-one tautomer **6b** of CA-M11.

Our previous findings have instilled confidence for further investigations around CA, emerged as the most promising candidate to use as a drug carrier, due to its favorable interaction profile with cL-BABP. Moreover, CA has shown well-documented merits, including its outstanding biocompatibility, appropriate bioavailability, rigid steroid backbone, minimal cytotoxicity, and distinctive amphiphilic characteristics^41^. Additionally, several studies report the synthesis and the biophysical and biological applications of CA-based conjugates, some of which exhibited antitumor activity, as already mentioned^37,42,43^. For building up a bioconjugate with CA endowed with anticancer activity, Mirin (**5**, Fig. 1), an inhibitor of DDR processes, was selected. Mirin has been deeply investigated, underlining interesting anticancer activities alone and in combination with cytotoxic drugs^21,28,30^. Indeed, its ability to act as chemo- and radiosensitizer has gained increasing attention over time but its use has never been approved due to the lack of favorable pharmacokinetics and high off-target toxicity. To address these drawbacks, we decided to link Mirin to CA by generating an ester moiety between the carboxylic acid of CA and the phenolic group of Mirin, finally achieving the structure of a novel conjugate, **CA-M11** (Fig. 5).

**Fig. 6:**
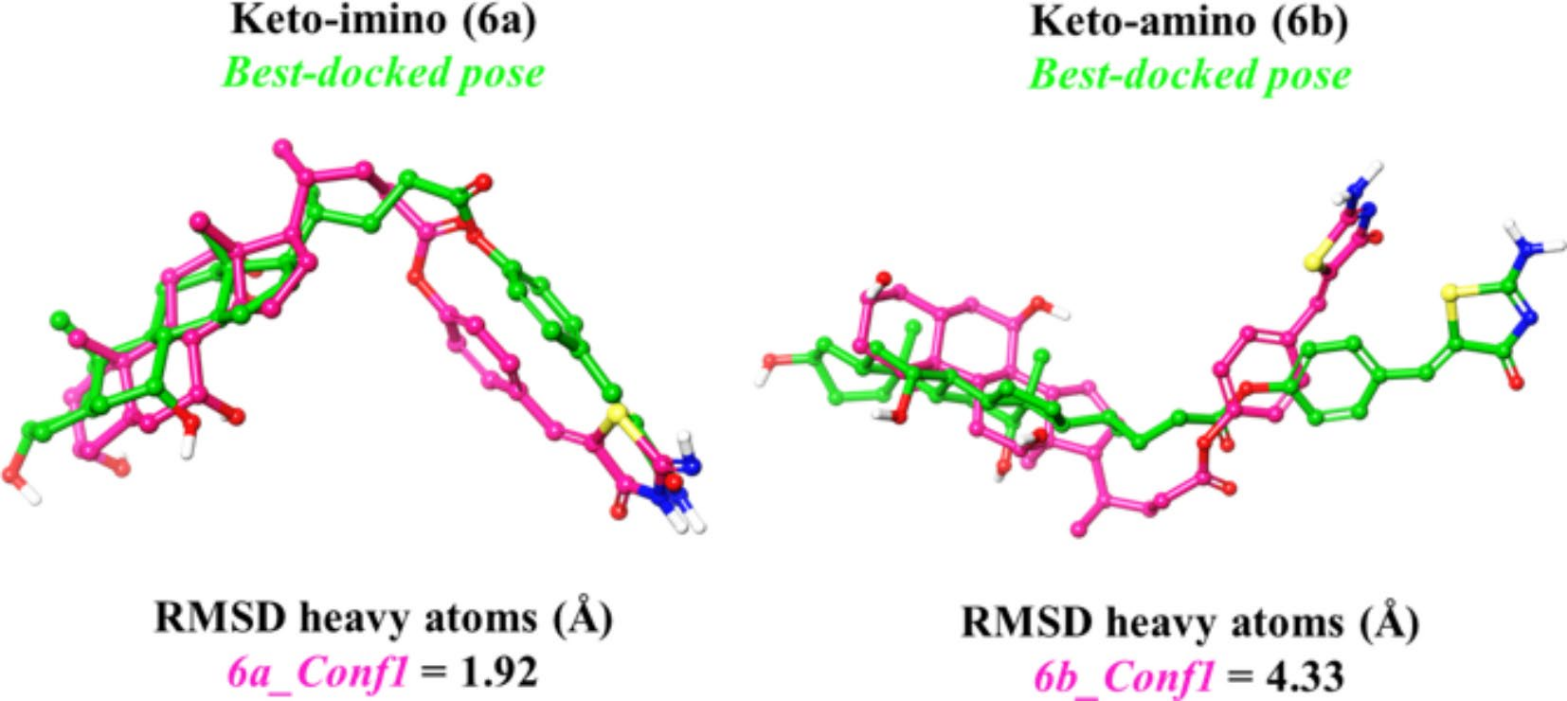
Geometrical comparison between the lowest-energy conformer of 6a and 6b (magenta carbon) and their respective best docked poses (green carbon). RMSD heavy atoms, expressed in Å, is provided.

Initially, we evaluated the likelihood of different tautomeric states and conformations for the bioconjugate. Indeed, it was necessary to take into account that CA-M11 contains an iminothiazolidin-4-one ring (**6a**) that may exist in a second aminothiazolidin-4-one tautomeric state **6b** (keto-imino–keto-amino type tautomerism). Iminothiazolidin-4-one is recognized as the predominant tautomer^29^. However, prior to conducting docking simulations, Monte Carlo conformational searches were performed on both tautomers **6a** and **6b**, to ensure confidence in the prediction between bound and unbound tautomeric **6a** and **6b** states in CA-M11. DFT optimizations were executed in the gas phase on the resulting conformations using Jaguar v 8.09. Then, higher level M06-2X/cc-pVTZ(-f) single-point energy (SPE) calculations were then carried out on the optimized conformations in gas and solution phase. The most stable conformations of **6a** and **6b** are depicted in Figure S2 (SI), along with their relative energies. Among these, **6a** emerged as the most stable state, exhibiting a preference for the keto-imino group, with energy disparities of approximately 7 and 3 kcal/mol in gas and solution phases, respectively. Following this molecular docking simulations were conducted to elucidate the interactions facilitating the accommodation of both tautomers of CA-M11 into cL-BABP. Upon comparing the calculated conformers of **6a** and **6b** with their respective best predicted docked poses, it became evident that the keto-imino (**6a**) conformation closely mirrored the one observed following recognition within the cL-BABP pocket (Fig. 6).

**Fig. 7:**
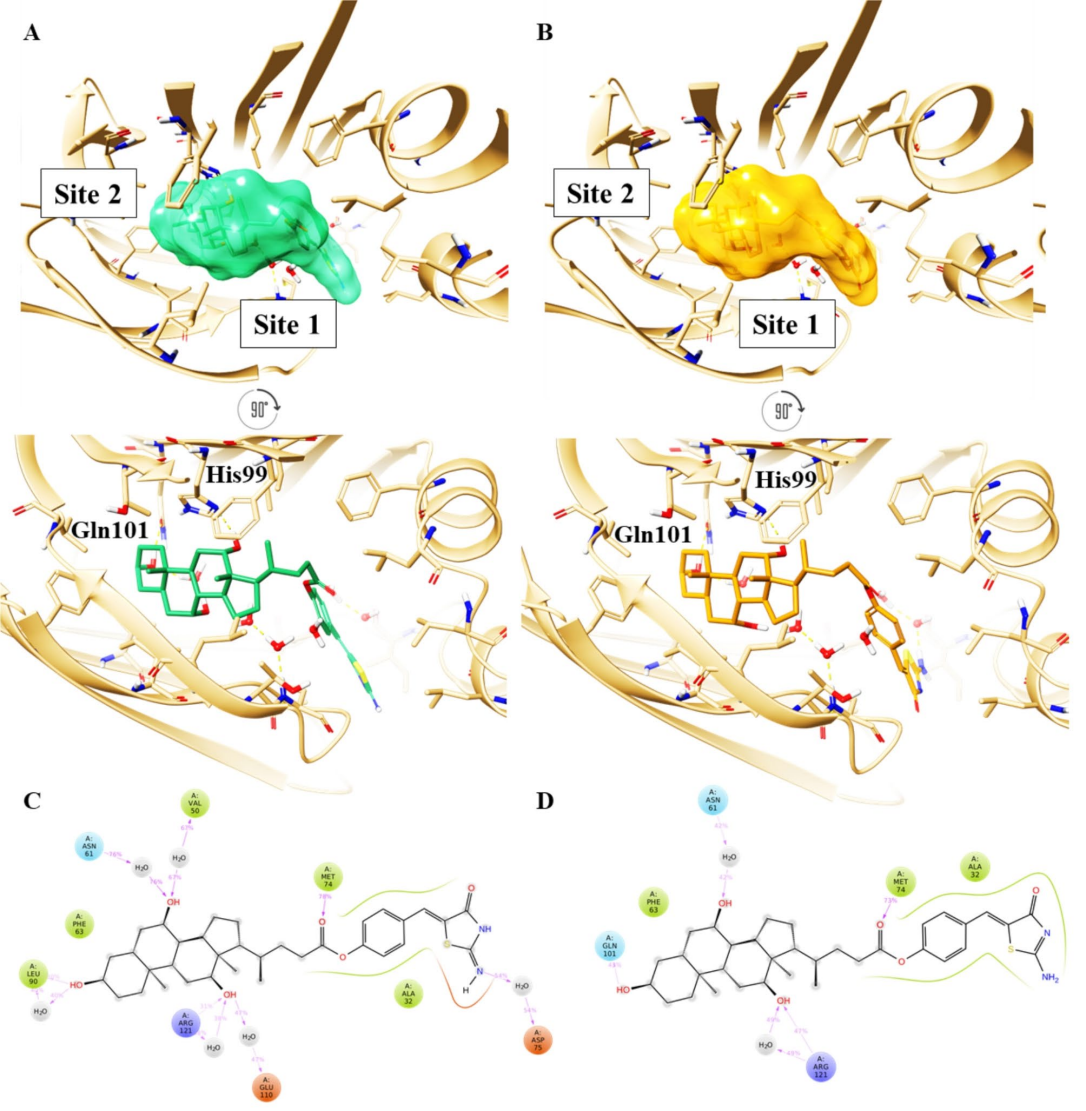
Top and front view of the best docking pose of (**A**) keto-imino (green carbon sticks) and (**B**) keto-amino tautomers (orange carbon sticks) of CA-M11 in complex with cL-BABP. The protein is reported as crème cartoon, with the residues involved in pivotal contacts shown as carbon sticks. The H-bonds are interactions are indicated as yellow dashed lines. Schematic representation of ligand-protein interactions of (**C**) keto-imino and (**D**) keto-amino tautomers of CA-M11 into cL-BABP. Interactions occurring more than 30% of the MDs are reported.

The top-scored docking poses were reported in Fig. 6. For the keto-imino tautomer, it was observed that the steroid skeleton of CA-M11 established an H-bond network with His99 and Gln101 (Fig. 7A). The keto-amino tautomer likewise located its CA portion in site 2 and its Mirin part towards site 1, showing a total flip of the amino-thiazolone ring compared to that of the keto-imino form (Fig. 7B). The best-docked conformation for each tautomer of CA-M11 into cL-BABP was submitted to 500 ns of MDs. Analyzing the contacts between the ligand and the protein, the keto-amino tautomer during the whole trajectory, it was observed that the hydroxyl at position 3 interacted with Leu90, while the hydroxyl groups at positions 7 and 12 engaged water bridges with Val50, Asn61, and Glu110, as well as Arg121, respectively. Since the portion bearing Mirin was exposed to the solvent, the imino group formed a water bridge with Asp75 for 54% of the MDs (Fig. 7C). The timeline chart of the keto-amino tautomer of CA-M11 showed that the contact with Gln101 was maintained for more than 40% of the MDs run. Similarly, the hydroxyl groups at positions 7 and 12 of CA-M11 interacted with Asn61 and Arg121, respectively, through a water bridge and H-bond. The carbonyl group was anchored to the Met74 residue for 73% of the MDs. Throughout the trajectory, several hydrophobic interactions were established with Ala 32 and Phe63 for both tautomers (Fig. 7D). Post-processing 500 ns of MDs, the binding free energy behavior was evaluated for both tautomers in complex with cL-BABP. Although they exhibited a similar energy profile, average values of -100.80 and − 98.56 kcal/mol were observed for keto-imino (**6a**) and keto-amino (**6b**) forms when bound to cL-BABP, respectively. This suggests a more stabilizing effect of **6a** compared to **6b**.

Therefore, Ab initio calculations determined that the lowest-energy tautomer and conformer for CA-M11 was the keto-imino form. Analysis of the MDs trajectory revealed that the interaction with Gln101 and Glu110 persisted throughout the simulation, indicating a stable binding mode of CA-M11 within the protein cavity. Specifically, the keto-imino form closely matches the bioactive pose obtained from the experimental assay (Figure S3, SI). It was confirmed that the steroid portion of CA-M11 occupies site 2 of cL-BABP, while the section containing Mirin is situated at site 1 of the cavity, with the iminothiazolidinonic ring (**6a**) directed towards the solvent-accessible area.

### Synthesis and characterization of mirin and CA-M11

**Scheme 1:**
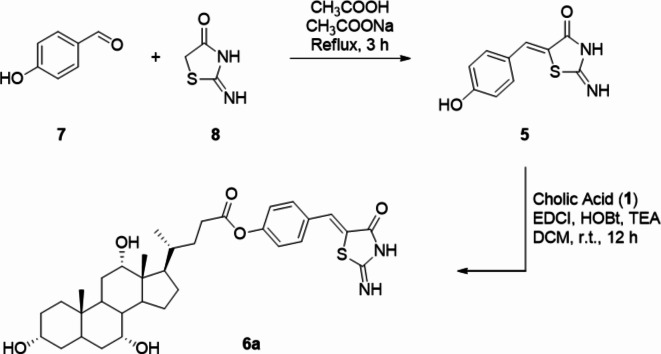
Synthesis of Mirin (**5**) and Cholic acid-Mirin bioconjugate CA-M11 (**6a**). Reagents and conditions are specified in the scheme.

The carrier-linker conjugation has been accomplished by generating an ester moiety on the carboxylic acid of CA using the phenolic group of Mirin (**5**). The synthesis of Mirin followed procedures outlined in existing literature^28–30^, as summarized in Scheme 1. By heating at reflux 4-hydroxybenzaldehyde (**7**) and 2-iminothiazolidin-4-one (**8**) in a mixture of acetic acid and sodium acetate, the Knoevenagel condensation afforded compound **5**. Mirin was then coupled with CA using EDCI and HOBt as condensing agents, in the presence of TEA as the base, resulting in the formation of CA-M11 (**6**) in moderate yield. NMR studies, LC-MS, and HRMS analyses confirmed the purity and quality of title compounds.

### Crystal structure of cL-BABP in complex with CA-M11

**Fig. 8:**
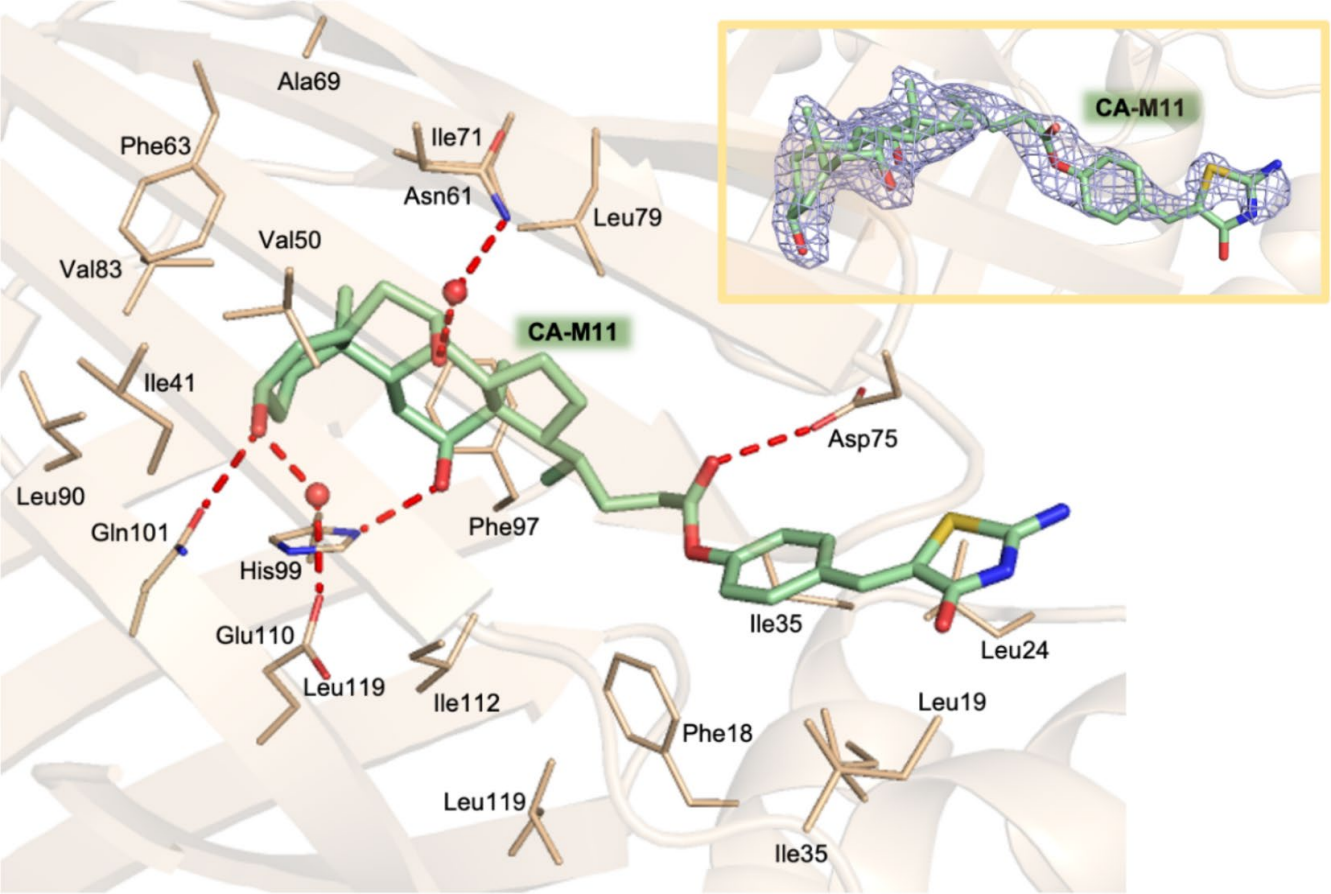
Ligand-binding cavity of cL-BABP in complex with CA-M11 (in sticks, light green carbons). The fitting of CA-M11 in the omit map (light blue, contoured at the 2.5 σ level) is shown in the inset.

The structure of cL-BABP in complex with CA-M11 was determined to 2.0 Å resolution (Table S1) having three protein chains in the asymmetric unit. CA-M11 fully populates the ligand-binding pocket in two chains (chains A and C of our model) while in the third (chain B) the electron density is too poor to model the ligand. However, CA-M11 in chain C was completely modeled whereas in chain A only the steroid rings and part of the aliphatic chain connecting CA to Mirin were modeled. The part of the molecule containing the steroid rings is accommodated in site 2 of the cavity and is involved in the same interactions described previously for the structure in complex with CA^35^ (Fig. 8). Its hydroxyl group at position 3 forms an H-bond with Gln101 and a water-mediated interaction with Glu110. The hydroxyl moiety 12 interacts with His99. The CA-M11 hydroxyl at position 7 forms a water-mediated interaction with Asn61. The carbonyl group on the ester functionality interacts with Asp75. The portion of the molecule containing Mirin is located at site 1 of the cavity and participates in van der Waals interactions with the surrounding residues. Accordingly, X-ray structural characterization validated the in silico predictions on CA-M11.

### Early ADME properties evaluation on CA-M11

**Fig. 9:**
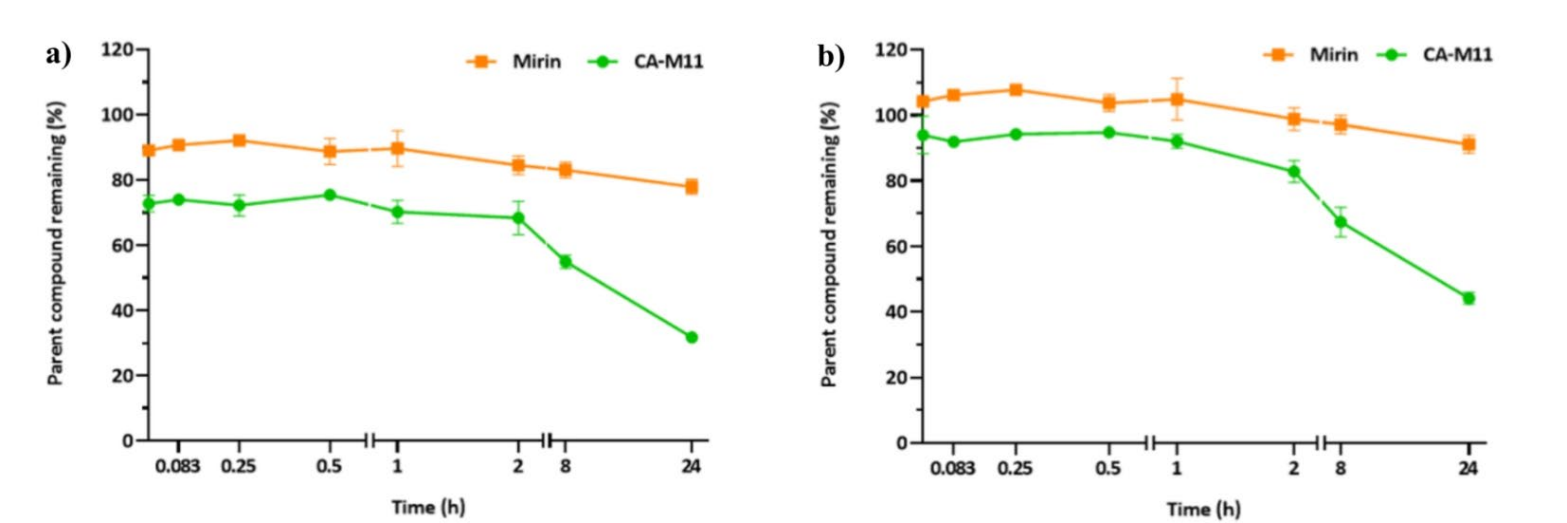
Stability profiles of compounds CA-M11 (red), and Mirin (blue) obtained in plasma (**a**) and DMEM 10% FBS (**b**). The values are reported as mean ± SD on *n* = 3 experiments run in triplicate. The ability of CA-M11 and Mirin to resist the metabolizing action of human liver microsomes was also investigated. Mirin maintained a significant stability after 1 h of incubation (90.65 ± 0.83%) leading to a low formation of the oxidized product M_2_ (1.52 ± 0.17%) (see Table 3).

To better elucidate the ADME properties of CA-M11 and Mirin, both compounds were incubated at a fixed concentration in the presence of human plasma for several time points to investigate the kinetics of hydrolysis. As shown in Table 2; Fig. 9, Mirin exhibited higher plasma stability over time, with a half-life (t_1/2_) greater than 24 h (96 h precisely). In fact, the percentage of unmodified compound never dropped below 80% up to 8 h of incubation, with only a slight decrease in stability observed after 24 h, reaching 77.94% of unmodified compound. In contrast, CA-M11 suffered more significant degradation even at the earliest time points. Approximately 30% of the compound was hydrolyzed by plasmatic esterases after a few minutes. The percentage of unmodified CA-M11 remained constant for up to 1 h of incubation (70.27%), but decreased steadily from 2 h onwards, stabilizing at around 68%. A significant decrease in plasma stability was observed after 8 h (54.99%) and 24 h (31.75%). Based on the percentage of plasma stability, the calculated half-life (t_1/2_) for CA-M11 was about 18 h. Then, the stability of both compounds in the presence of Dulbecco’s Modified Eagle Medium (DMEM) added with 10% FBS, 1% PS, and 1% Glu was investigated, with experiments conducted at the same time points for better comparison with the results on plasma stability.

**Table 2:** Plasma and DMEM cell culture medium stability of Mirin and CA-M11. The values are reported as mean ± SD on *n* = 3 experiments run in triplicate.

	Plasma stability (% ± SD)		DMEM cell culture medium stability (% ± SD)	
Time (h)	Mirin	CA-M11	Mirin	CA-M11
**0**	89.10 ± 1.03	72.79 ± 2.57	104.19 ± 1.20	93.96 ± 5.76
**0.083**	90.80 ± 0.13	74.01 ± 0.65	106.18 ± 0.16	91.94 ± 0.85
**0.25**	92.18 ± 1.34	72.29 ± 3.22	107.78 ± 1.57	94.24 ± 0.37
**0.5**	88.72 ± 3.98	75.47 ± 0.04	103.73 ± 2.61	94.77 ± 0.60
**1**	89.70 ± 2.23	70.27 ± 3.49	104.88 ± 6.37	92.08 ± 2.15
**2**	84.51 ± 2.93	68.45 ± 5.08	98.81 ± 3.43	82.83 ± 3.38
**8**	83.07 ± 2.40	54.99 ± 2.07	97.14 ± 2.81	67.45 ± 4.54
**24**	77.94 ± 2.30	31.75 ± 1.51	91.14 ± 2.70	44.41 ± 1.76
**t**_**1/2**_^**a**^	96.3	18.1	113.6	21.7

^a^Half-life (h) expressed as the amount of time it takes before half of the drug is hydrolyzed/degraded

As indicated in Table 2 and illustrated in Fig. 9, no significant degradation was detected at the earliest time points for both compounds incubated with the DMEM cell culture medium. Mirin exhibited stability percentages consistently above 90%, resulting in a half-life (t_1/2_) value greater than 100 h. CA-M11 resulted very stable until 1 h of incubation, after which the percentage of unmodified compound gradually decreased, reaching 67.57% at 8 h. Finally, the percentage of stability collapsed at 44.41% at 24 h. However, the half-life value of CA-M11 resulted in 21.7 h.

**Table 3:** Metabolic stability assay for Mirin and CA-M11.

Compounds	Metabolic stability (%)		Metabolite formation (%)^a^	
	Time (min)		Time (min)	
	0’	60’	0’	60’
**Mirin**	92.32 ± 1.93	90.65 ± 0.83	-	M_2_ = 1.52 ± 0.17
**CA-M11**	16.46 ± 1.41	5.34 ± 0.58	M_1_ = 53.48 ± 4.08	M_1_ = 87.52 ± 4.50 M_2_ = 0.94 ± 0.07

^a^M_1_= Mirin released after ester bound breakage. ^b^M_2_ = M_1_ + OH (+ 16). Data are reported as the mean ± standard deviation (SD) of *n* = 3 experiments run in triplicates

On the other hand, CA-M11 conjugate was very labile in the presence of hepatic microsomes. In fact, starting from 0 min, this compound underwent substantial metabolic processes, releasing the active component, Mirin. The percentage of non-metabolized CA-M11 was already below 20% at the beginning of the experiment and continued to decrease over time, settling at around 5% after one hour of incubation (Table 3). Consequently, it is not surprising to observe high percentages of Mirin released from CA-M11 (M_1_ = 53.48 ± 4.08%) at the beginning of the assay, with its formation rate increasing over time (M_1_ = 87.52 ± 4.50% after 1 h). Furthermore, a small percentage of the oxidized metabolite M_2_ could also be identified after 1 h of incubation (0.94 ± 0.07%). Since the sum of percentages of parent compound (CA-M11 or Mirin), M_1_ (Mirin released from CA-M11) and M_2_ (the oxidized Mirin) did not result in 100%, we hypothesized that some complexation phenomena might have occurred among the compounds and microsomes. Therefore, matrix effects were investigated by preparing fresh time-zero solutions made of denatured microsomes, solvent mix and tested compounds. To have a better correlation with the time-zero of the metabolic stability experiments, final volumes and concentrations used were not modified. The chromatographic analysis (Table S2, SI) revealed that both compounds engaged in binding phenomena with microsomes. Indeed, percentages of extraction recovery (% Rec) resulted very high for Mirin which was characterized by a percentage of interaction lower than 10% (% Rec 92.32 ± 1.92), but CA-M11 demonstrated a significant affinity towards microsomes with % Rec lower than 70%. These data justified the “loss of material” detected in the metabolic stability assay and supported the hypothesis that some interactions may occur between compounds (especially CA-M11) and microsomes. As a matter of fact, CA-M11 displayed a substantial stability in plasma, and a similar trend was observed in the cell culture medium. The slight increase of stability and t_1/2_ in the cell medium may be due to the lower amount of proteins present with respect to plasma. Mirin possessed an analogous trend only with higher percentages of stability in both tests. Notably, while Mirin remained significantly stable under the oxidative conditions of hepatic microsomes, CA-M11 underwent rapid metabolism, leading to the release of Mirin. On the one hand, this could be considered a stability issue for CA-M11, however, on the other hand, it contributes to rapidly release Mirin in the proper compartment, the liver, where the microsomes exert their maximum activity. Additionally, permeability studies were conducted to investigate the ability of both tested compounds to passively cross phospholipid bilayers. When Mirin and CA-M11 were incubated in the presence of an artificial phospholipidic membrane, their ability to cross the bilayer by exploiting passive diffusion mechanisms resulted quite limited. Indeed, apparent permeability (P_app_) values minor than 0.1 × 10^−6^ cm s^−1^ resulted for both compounds (Table S3, SI), underlining no significant differences among their ability to diffuse in the acceptor compartment exploiting passive mechanisms. Moreover, the PAMPA assay showed poor interactions between tested compounds and the cell membrane, as indicated by the low percentages of membrane retention (MR%), suggesting that compounds did not remain entrapped into the phospholipidic bilayer. The limited passive diffusion across cell membranes for both CA-M11 and Mirin is in line with the presence of CA, as it is reported that both free and conjugated BAs cannot cross membranes by passive diffusion, but they usually require some active transports in and out of cells. In this process, BAs are usually transported by ATP-binding protein transporters which actively allow their passage across cellular membranes^44^. Therefore, the lack of significant passive permeability for CA-M11 may suggest that it exploits active transport mechanisms to reach the intracellular target.

### In vitro cytotoxicity

Along with early ADME properties, CA-M11 has been thoroughly investigated to characterize its potential anticancer activity on HepG2 cells, used as an in vitro model of liver cancer. The results obtained with CA-M11 were compared to those collected in the same tests with Mirin alone. In the biological evaluation, not only the effect of the conjugate by itself was investigated, but also its effect in combination with a cytotoxic agent whose mechanism of action also encompasses DNA-damage induction was assessed. Doxorubicin, one of the most used cytotoxic agents, with high percentages of responders^45^, was selected for this aim.

**Fig. 10:**
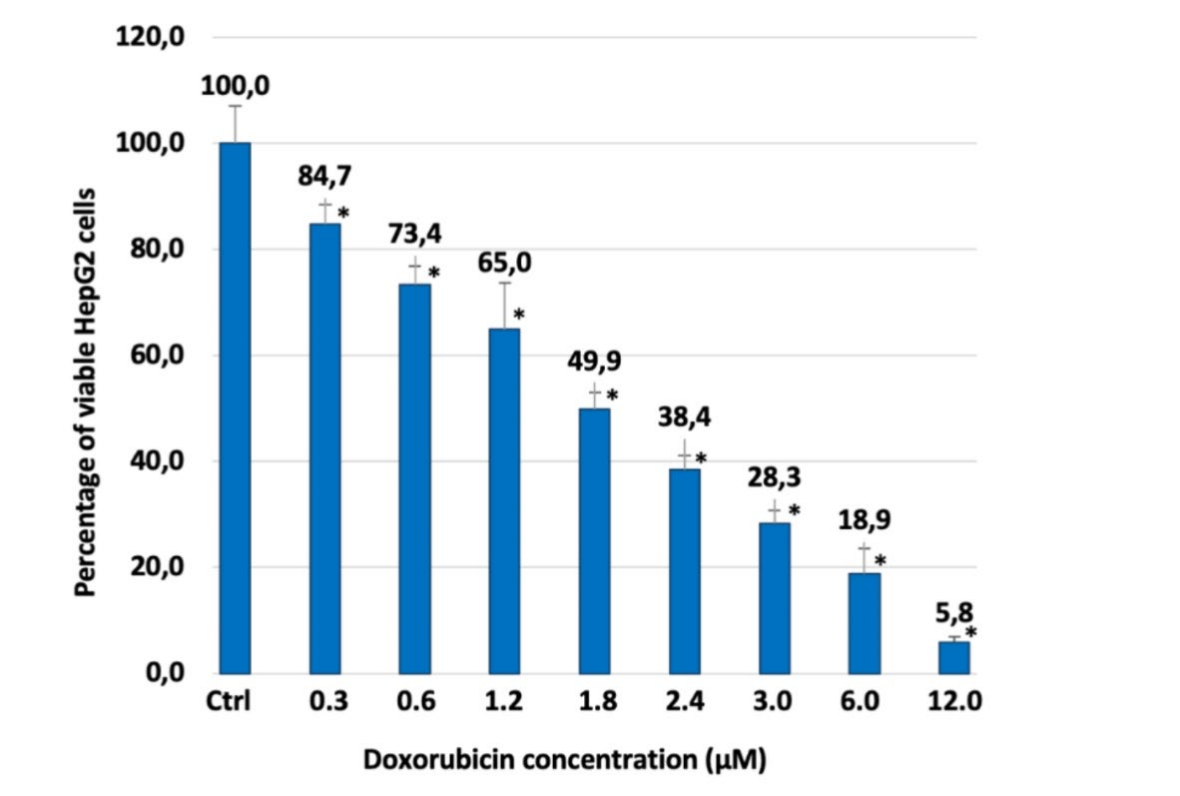
Percentage of viable HepG2 cells after 24 h of contact with different concentrations of Doxorubicin, as determined by the Neutral Red Uptake. Data are mean ± SD of three experiments run in six replicates. *Values are statistically different versus negative control (complete medium, i.e. concentration 0 µM), *p* < 0.05.

**Fig. 11:**
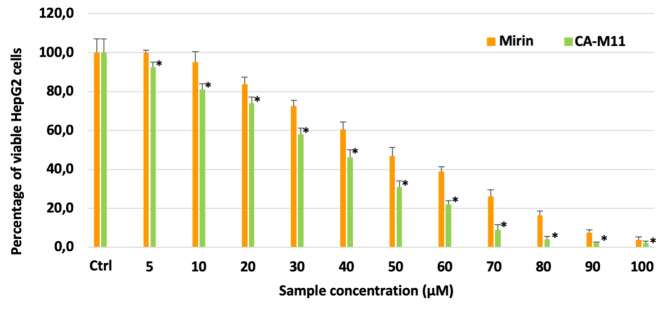
Percentage of viable HepG2 after 24 h of contact with different concentrations of Mirin and CA-M11, as determined by the Neutral Red Uptake. Data are mean ± SD of three experiments run in six replicates. *Values are statistically different versus Mirin 5 µM, *p* < 0,05.

**Fig. 12:**
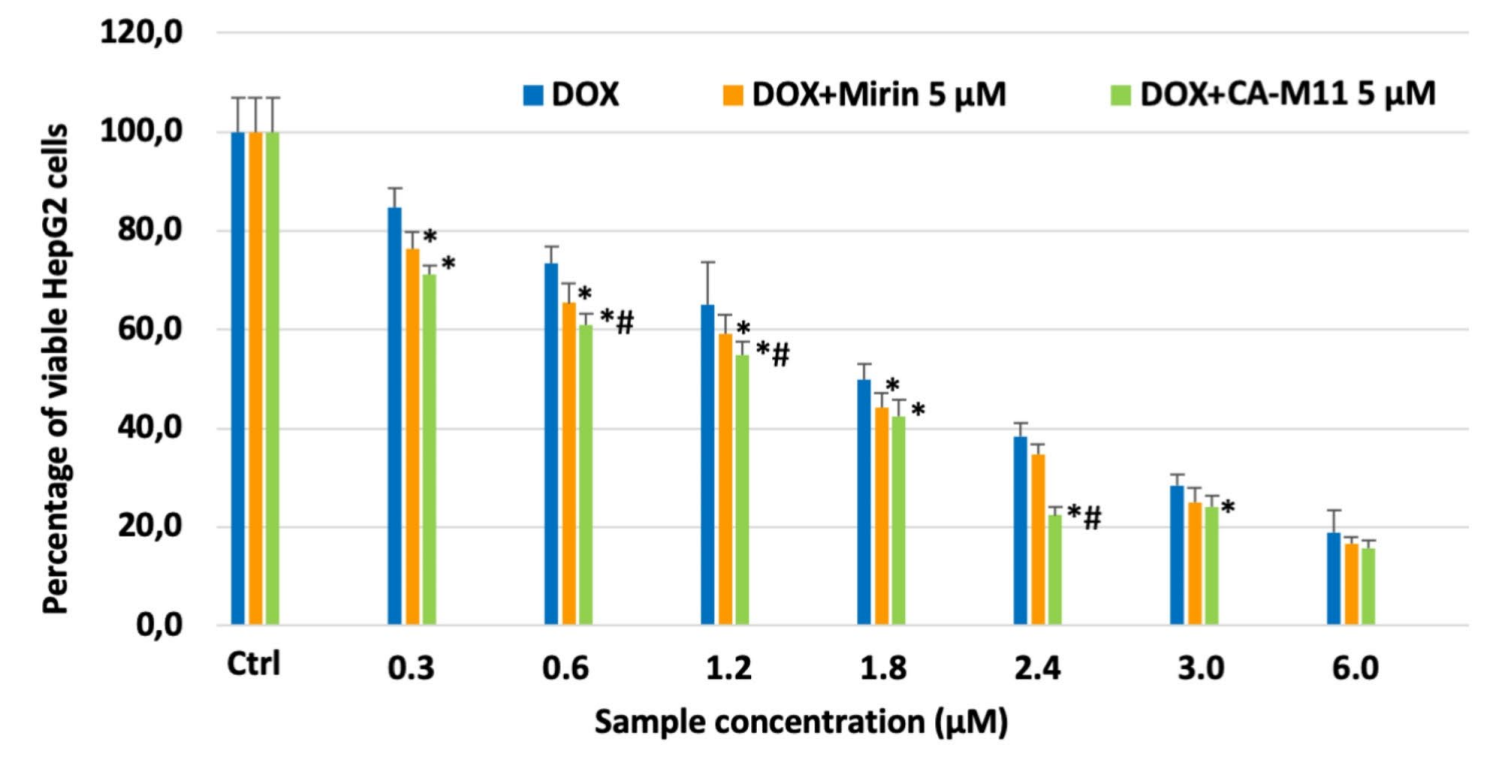
Percentage of viable HepG2 after 24 h of contact with different concentrations of Doxorubicin (DOX), and Doxorubicin + Mirin 5 µM and Doxorubicin + CA-M11 5 µM, as determined by the Neutral Red Uptake. Data are mean ± SD of three experiments run in six replicates. *Values are statistically different versus Doxorubicin, *p* < 0.05; # Values are significantly different from DOX-Mirin 5 µM, *p* < 0,05.

Non-confluent adhered HepG2 cells were incubated with different concentrations of Doxorubicin, Mirin, and CA-M11. Following a 24 h incubation timeframe, cells were analyzed, and the results are reported in Figs. 11 and 12. As shown in Fig. 10, the cytotoxic effect of Doxorubicin increased in parallel with its concentration, exhibiting an IC_50_ value of 1.8 µM. Mirin and CA-M11 also demonstrated a cytotoxic effect that rose with increasing concentrations, displaying IC_50_ values of 50 µM and 40 µM, respectively (Fig. 11). For all concentrations tested, CA-M11 demonstrated significantly greater cytotoxic activity against HepG2 cells than Mirin. Figure 12 reports the percentage of viable HepG2 cells after 24 h following treatment with increasing concentrations of Doxorubicin co-administered with a 5 µM concentration of Mirin or the same concentration of CA-M11. Notably, the presence of Mirin and CA-M11 enhanced the toxic effect of Doxorubicin on HepG2 cells in a concentration-dependent manner. This enhancement was observed for Doxorubicin concentrations ranging from 0.3 to 1.8 µM in the presence of Mirin, and from 0.3 to 3 µM in the presence of CA-M11. Furthermore, the addition of 5 µM CA-M11 increased the cytotoxic effect of Doxorubicin significantly more than Mirin.

For the sake of data completeness, mutagenicity evaluation in *Salmonella typhimurium* strains TA98 and TA100 was also performed. Doxorubicin, Mirin and CA-M11 showed a mutagenic effect starting from 10 µM which increases by raising their concentrations on both TA98 and TA100 strains (Figure S4, SI). The presence of Mirin and CA-M11 was not able to affect Doxorubicin mutagenicity for both TA 98 and TA 100 strains (Figure S5, SI). However, the cytotoxic effect of Doxorubicin was potentiated by the concomitant treatment of CA-M11, in a dose-dependent manner. This was particularly evident for Doxorubicin concentrations ranging from 0.6 to 2.4 µM, with the maximum effect happening at 2.4 µM, where a 5 µM concentration of CA-M11 increased cell death of roughly 40%. The administration of an equal concentration of Mirin did not achieve the same effect of CA-M11, although significantly increasing cytotoxicity in comparison with Doxorubicin alone. This might correlate to differences in cell penetration for the two agents. The biological results obtained with our bioconjugate are definitely worth of note, since the tested concentration of CA-M11 is much lower than its IC_50_ value. Actually, at 5 µM, CA-M11 does not exert a significant cytotoxic effect on HepG2 cells (Fig. 11), while the same concentration is able to significantly potentiate the effect of the cytotoxic agent Doxorubicin (Fig. 12). Therefore, the lack of cytotoxicity at doses as low as 5 µM combined with the chemo-sensitizing effect induced on Doxorubicin treatment represents a real opportunity to find renewed application for Mre-11 inhibitors as bioconjugates with CA. Worth of note, our preliminary results could also be exploited to set up future experiments in 3D spheroids models and in vivo studies. Mirin has already been tested in ovarian cancer cells (A2780cis and PEO4) and in 3D spheroid (A2780 and HeLa cells), proving to be efficacious in reverting platinum resistance^21^. Hence, this highlights the potential of our strategy to be translated to more complex in vitro^46,47^ and in vivo models^48^.

## Conclusions

In conclusion, in silico studies guided the selection of CA as the preferred BA for the interaction with cL-BABP, emerging as the most promising carrier for a selected Mre11 inhibitor, namely Mirin. This latter is renowned for its anticancer activity, albeit hindered by inadequate pharmacokinetic properties and significant off-target toxicity. For the first time, it has been demonstrated the successful accommodation of a CA-based bioconjugate with Mirin (CA-M11) within the ligand-binding cavity of cL-BABP, as marked by the analysis of the crystal structure of the complex, which provides strong evidence that our bioconjugate can serve as a substrate for the protein. As aforementioned, the similarity between cL-BABP and FABPs allows to speculate about the interaction of our conjugate with the mammal proteins and its trafficking in the districts of interest. These preliminary yet encouraging findings demonstrate the potential of our approach in developing novel conjugates as adjuvant treatments for liver cancer. In fact, Mirin, delivered by CA-M11 in the hepatic compartment where BAs accumulates, can inhibit DDR processes, leading to the possibility of reducing the dose of Doxorubicin while achieving an equivalent antitumor effect. Moreover, considering that the cytotoxic agent is an Mre11 inhibitor, our findings also indicate this strategy as a promising opportunity for a renewed application of DDR inhibitors in cancer therapy. Our strategic design paves the way for the exploitation of iLBPs in the transport of bioactive substances to the liver. In addition, we can reach out for the creation of different analogues of CA-M11, possibly bearing different anticancer agents or other drugs which has to be directed to the liver, thanks to the sensor-activity of CA or other BAs as carriers. Following, investigation will be performed moving from the in vitro to the in vivo evaluation, to further elucidate the activity and metabolic fate of BAs-bearing bioconjugates.

## Experimental section

### Computational studies

All in silico studies were performed by using Schrödinger Suite 2021 − 4.^49^ The X-ray crystallographic structure of the cL-BABP in complex with CA, deposited in the Protein Data Bank (PDB code: 7O0K)^35^, was prepared by means of the Protein Preparation Wizard tool^50^. At pH 7.4, the hydrogen atoms were added, and the energy minimization calculation was carried out adopting the OPLS_2005 force field^51^ until the RMSD of all heavy atoms within 0.3 Å. The 3D structures of CDCA, DCA, LCA, and UDCA were generated with LigPrep 5^52^, which allowed ligand preparation by adding hydrogen atoms, neutralizing charged groups and generating the different ionization states and possible tautomers followed by energy minimization using the same force field previously used for the BABP structure preparation. Docking calculations of BAs against BABP protein were performed with Glide^53^. The standard precision (SP) mode was run using default settings and 10 poses for each investigated BA were computed. Docking box grids of about 27,000 Å3 were centered on the co-crystallized bioactive poses to define both binding sites, named site 1 and site 2. The GlideScore function was adopted as criteria to select the best BA into the pocket. Indeed, this score aims to effectively distinguish compounds with high binding affinity from those with low or no binding ability. As an empirical scoring function, it incorporates several terms that reflect the physics of the binding process. These include a lipophilic-lipophilic interaction term, terms for hydrogen bonding, a penalty for rotatable bonds, and contributions from protein-ligand coulomb and van der Waals energies. Additionally, GlideScore accounts for hydrophobic enclosure, which is the displacement of water molecules by a ligand in regions surrounded by many nearby lipophilic protein atoms. According to the GlideScore function, for each complex, the best docked pose was submitted to 500 ns of molecular dynamic simulations (MDs) by using Desmond software ver. 4.2^54^. Each simulation was carried out under the following conditions: recording interval equal to 500 ps; 500 ns of simulation time at 300 K; pressure set to 1 bar; OPLS_2005 as force field. The systems were solvated in the TIP3P explicit solvent model^55^, and the counter ions were added to neutralize the system’s net charge. MDs analyses were performed through the “Simulation Interactions Diagram” for monitoring protein − ligand interactions. Subsequently, the MM/GBSA method^56,57^ was applied to estimate the ligand-binding affinity and to identify which BA interacts most effectively with BABP. The calculation relies on the following equation:

$$\Delta G{\rm{bind}} = G{\rm{comp}} - G{\rm{rec}} - G{\rm{lig}} = \Delta E{\rm{ele}} + \Delta E{\rm{vdW}} + \Delta E{\rm{int}} + \Delta E{\rm{GB}} + \Delta E{\rm{surf}}$$

where Gcomp, Grec, and Glig denotes the free energy of the complex, the receptor, and the ligand, respectively. The total energy takes account of the single energy contribution of the electrostatic energy term (ΔEele), the van der Waals energy term (ΔEvdW), and the bond, angle, and dihedral term (ΔEint) in the gas-phase condition. ΔEGB and ΔEsurf indicate the polar and nonpolar desolvation free energy, respectively. The implicit solvation was calculated using the GB model^58^ while the non-polar solvation energy was calculated using the solvent-accessible surface area algorithm. The entropic term was neglected, due to the limitations in the entropy calculation method, a common approximation^59^. After the identification of the best BA in complex to BABP, the ligands i.e. Mirin and the conjugate CA-M11 were built for further investigation. For the tautomeric prediction of CA-M11, we used the QM Conformer & Tautomer Predictor tool of Maestro utilizing Jaguar^60^ in the quantum mechanical (QM) calculations. The initial step involves identifying the proton donor and acceptor atoms within the molecule, followed by redistributing protons among these atoms to generate a range of tautomers. Subsequently, the tautomers are assessed based on their semiempirical PM3 heat of formation, with high-energy tautomers being eliminated. The surviving tautomers then undergo conformational analysis using MacroModel to generate a set of conformers. High-energy conformations are filtered out based on their semiempirical PM3 heat of formation. Following this, the lowest-energy tautomers undergo DFT geometry optimizations using the B3LYP-D3/LACVP** level of theory, and the obtained geometries were ranked computing single–point energies at the M06-2X/cc-pVTZ(-f) level of theory. Thus, docking simulations were performed on the top conformers for the lowest-energy tautomeric forms of CA-M11. For each of them, the best docked pose was subjected to 500 ns of MDs using the same aforementioned procedure.

### Synthetic procedures for compounds 5 and 10

*General information*. All reagents and solvents were purchased from Merck and were used as received. Merck silica gel 60 (230–400 mesh) was used for column chromatography. Merck aluminum sheets coated with silica gel 60 F254 were used for TLC. 1H spectra were recorded with a Bruker DRX 600 AVANCE spectrometer in the indicated solvent (the residual solvent peaks were used as the internal standard). The values of the chemical shifts (δ) are reported in ppm, and the H–H coupling constants (*J*) are expressed in Hz. An Agilent 1100 LC-MS running with an electrospray source (ESI) was used in mass spectrometry measurements. The purity of compounds **5** and **10** was assessed by LC-MS analysis and was found to be higher than 95%. An Infinity Lab Poroshell 120 EC-C18 column 2.1 × 50 mm, 2.7 μm was used as the stationary phase while a 5–95% gradient of MeCN (+ 0.1% HCOOH) in H_2_O (+ 0.1% HCOOH) in 5 min represented the mobile phase. UV detection was performed at 254 nm. A Bruker Tims-TOF instrument with an ESI source was used to measure the high-resolution mass (HRMS) values.

*(Z)-5-(4-Hydroxybenzylidene)-2-iminothiazolidin-4-one (5)*. 4-Hydroxybenzaldehyde (**11**, 1.0 g, 8.2 mmol) and 2-iminothiazolidin-4-one (**12**, 1.1 g, 9.0 mmol) were heated at reflux in a mixture of NaOAc (2.0 g, 24.6 mmol) and AcOH (10 mL). The resulting orange solid was filtered and washed several times with cold water. The pure compound was obtained as an orange solid (1.4 g, 78% yield) with no further purification. 1H NMR (600 MHz, CD_3_OD) δ 7.64 (s, 1 H), 7.43 (d, *J* = 8.6 Hz, 2 H), 6.90 (d, *J* = 8.7 Hz, 2 H). HRMS (ESI) m/z: [M + H]^+^ Calcd for C_10_H_9_N_2_O_2_S^+^ 221.03792, Found 221.03773; [M + Na]^+^ Calcd for C_10_H_8_N_2_NaO_2_S^+^ 243.01987, Found 243.01962 (Figure S6, ESI).

*(4R)-4-((Z)-(2-Imino-4-oxothiazolidin-5-ylidene)methyl)phenyl 4-((3R*,*7R*,*10 S*,*12 S*,*13R*,*17R)-3*,*7*,*12-trihydroxy-10*,*13-dimethylhexadecahydro-1 H-cyclopenta[a]phenanthren-17-yl)pentanoate (10*,* CA-M11)*. To a stirred solution of cholic acid (100 mg, 0.25 mmol) and Mirin (55 mg, 0.25 mmol) in dry THF (5 mL), EDCI (96 mg, 0.5 mmol) and DMAP (16 mg, 0.1 mmol) were added. The mixture was stirred at room temperature for 16 h. Volatiles were removed under reduced pressure and the crude was purified by means of chromatography on silica gel (EtOAc in PE gradient, 10:90 to 90:10) to afford pure compound **CA-M11** (45 mg, 30% yield) as a light yellow solid. LC-MS ESI *m/z*: *rt* = 1.527 min, 611.0 [M + H]^+^, 632.9 [M + Na]^+^, 609.0 [M-H]^−^ (Figure S7, ESI). HRMS (ESI) *m/z*: [M + Na]^+^ Calcd for C_34_H_46_N_2_NaO_6_S^+^ 633.29688, Found 633.29692 (Figure S8, ESI).

### Protein expression, purification, and crystallization

Recombinant cL-BABP was expressed and purified following the established protocol, with minor modifications^35^. Crystals of cL-BABP in complex with BAs and CA-M11 were obtained by co-crystallization using the sitting drop vapor diffusion technique at 8 °C^61^, according to the reported protocol^35^. Briefly, samples for the crystallization experiment were prepared by adding 5 mM compounds (dissolved in dimethyl sulfoxide, DMSO) to the protein solution (25 mg mL^−1^ in 100 mM sodium chloride and 20 mM TRIS, pH 7.5) and incubating 1 h at 4 °C. Crystallization drops, consisting of equal volumes of these samples and precipitant solution, were equilibrated over a 200 µL reservoir at 8 °C. For the cL-BABP-CDCA complex, the precipitant formulation was 200 mM sodium chloride, 25% w/ol PEG-3500 and 100 mM HEPES, pH 7.5, for cL-BABP-UDCA complex was 12% w/v PEG-3500 and 100 mM sodium formate, pH 7.50, while for cL-BABP-DCA and cL-BABP-LCA complexes was 25% w/v PEG-3500, 200 mM lithium sulfate monohydrate, and 100 mM BIS-TRIS, pH 6.5. The crystals of cL-BABP-CA-M11 complex grew in 2.4 M sodium malonate, pH 7.0. Prior to flash freezing in liquid nitrogen, crystals were singularly transferred to the cryoprotectant solution prepared by adding to each precipitant 20% v/v of PEG-400.

### Data collection, structure solution and refinement

Diffraction data were collected using synchrotron radiation at the Diamond Light Source (DLS, Didcot, UK) beamline I04 equipped with a Eiger2 XE 16 M detector. Crystals of cL-BABP in complex with the BAs belonged to the monoclinic space group P2_1_, whereas those in complex with CA-M11 to the orthorhombic space group P2_1_2_1_2_1_. Data were integrated using XDS^62^ and scaled with Scala^63,64^ from the CCP4 suite^65^. Data collection and processing statistics are reported in Table S1. Molecular replacement was performed using the software Molrep^66^ and the structure of cL-BABP in complex with CA (PDB id 7O0K^35^, excluding non-protein atoms and water molecules) was used as a searching model. All structures were refined through a combination of automatic refinement cycles using REFMAC5^67^ from the CCP4 suite^65^ and manual correction and rebuilding using the molecular graphic software Coot^68^. Water molecules were added through the program Coot and checked both manually and automatically. In all complexes, the inspection of the Fourier difference map clearly evidenced the presence of the compound inside the cavity, which was manually placed inside the ligand-binding cavity according to the electron density. Final models were inspected manually and checked with the programs Coot^68^ and Procheck^69^. Final refinement statistics are reported in Table S1. Figures were generated using Pymol^70^. Final coordinates and structure factors were deposited in the Protein Data Bank (PDB) under the codes 9ETE (cL-BABP-DCA), 9ETF (cL-BABP-LCA), 9ETD (cL-BABP-UDCA), 9ETC (cL-BABP-CDCA), and 9ETG (cL-BABP-CA-M11).

### Biological evaluation

Dulbecco’s Modified Eagle’s Medium, trypsin solution, and all the solvents used for cell culture were purchased from Lonza (Switzerland). Human hepatoma HepG2 cells were purchased from American Type Culture Collection (USA). The mutagenicity assay was supplied by Biologik s.r.l. (Trieste, Italy).

*Cell cultures and cytotoxicity assay*. To evaluate the in vitro cytotoxicity of the test samples, the direct contact tests, proposed by ISO 10995-5, Biological evaluation of medical devices – Part 5: Tests for cytotoxicity: in vitro methods were used^71^. This test is suitable for sample with various shapes, sizes, or physical status (i.e. liquid or solid). Among the recommended cells lines reported in the document ISO 10995-5:2009 to test Mirin, Doxorubicin, and CA-M11 cytotoxicity, Human hepatoma HepG2 cells were chosen.

*Evaluation of HepG2 viability.* Cells were maintained in DMEM at 37 °C in a humidified atmosphere containing 5% CO_2_. The culture medium was supplemented with 10% fetal calf serum (FCS), 1% L-glutamine-penicillin-streptomycin solution, and 1% MEM Non-Essential Amino Acid Solution. Once at confluence, cells were washed with PBS 0.1 M, taken up with trypsin-EDTA solution and then centrifuged at 1000 rpm for 5 min. The pellet was re-suspended in medium solution (dilution 1:15).

Cells (1.5 × 104) suspended in 1 mL of complete medium were seeded in each well of a 24 well round multidish and incubated at 37 °C in an atmosphere of 5% CO_2_. Once reached the 50% of confluence (i.e. after 24 h of culture), the culture medium was discharged and the test compounds, properly diluted in completed medium, were added to each well. The stock solutions of Mirin, Doxorubicin, and CA-M11 were prepared in DMSO. The following concentrations of Mirin and CA-M11 were tested: 5; 10; 20; 30; 40; 50; 60; 70; 80; 90 and 100 µM. The tested concentrations of Doxorubicin were 0.3; 0.6; 1.2; 1.8; 2.4; 3.0; 6.0 and 12.0 µM. Then, the experiments were repeated adding to Doxorubicin concentrations ranging from 0.3 to 6.0 µM, respectively, Mirin 5 µM, and CA-M11 5 µM. These values were chosen because of cytotoxic effect towards HepG2 cells. Each experiment was repeated 3 times, and all samples were set up in six replicates. A complete medium was used as negative control. After 24 h of incubation, cell viability was evaluated by Neutral Red uptake, as follows. First, the following solutions were prepared in order to determine the percentage of viable cells: (1) Neutral Red (NR) Stock Solution: 0.33 g NR Dye powder in 100 mL sterile H_2_O; (2) NR Medium: 1.0 mL NR Stock solution + 99.0 Routine Culture Medium pre-warmed to 37 °C); (3) NR Desorb solution: 1% glacial acetic acid solution + 50% ethanol + 49% H_2_O. At the end of the incubation, the routine culture medium was removed from each well, and cells were carefully rinsed with 1 mL of pre-warmed D-PBS. Multiwells were then gently blotted with paper towels. 1.0 mL of NR Medium was added to each well and further incubated at 37 °C, 95% humidity, 5.0% CO_2_ for 3 h. The cells were checked during the NR incubation for NR crystal formation. After incubation, the NR Medium was removed, cells were carefully rinsed with 1 mL of pre-warmed D-PBS. Then, the PBS was decanted and blotted from the wells and exactly 1 mL of NR Desorb solution was added to each sample. Multiwells were then put on a shaker for 20–45 min to extract NR from the cells and form a homogeneous solution. During this step the samples were covered in order to protect them from light. After 5 min from the plate shaker removal the absorbance was read at 540 nm by a UV/visible spectrophotometer (UV5, Mettler Toledo).

*Mutagenicity assay: Ames test.* The TA100 and TA98 strains of Salmonella Typhimurium were utilized for mutagenicity assay. Approximately 107 bacteria were exposed to 6 concentrations of each test compound, as well as a positive and a negative control, for 90 min in a medium containing sufficient histidine to support approximately two cell divisions. After 90 min, the exposure cultures were diluted in pH indicator medium lacking histidine, and aliquoted into 48 wells of a 384-well plate. Within two days, cells which had undergone the reversion to His grew into colonies. Metabolism by the bacterial colonies reduced the pH of the medium, changing the color of that well. This color change can be detected visually or by a microplate reader. The number of wells containing revertant colonies were counted for each dose and compared to a zero-dose control. Each dose was tested in six replicates. The following concentrations of Mirin and CA-M11 were tested: 5; 10; 30; 50; 80 and 100 µM. The concentration values tested of Doxorubicin were: 0.3; 1.2; 1.8; 3.0; 6.0 and 30 µM. The experiments were then repeated adding to Doxorubicin concentrations ranging from 0.3 to 30 µM, respectively, Mirin 5 µM and CA-M11 5 µM.

*Statistical analysis*. Multiple comparison was performed by one-way ANOVA and individual differences tested by Fisher’s test after the demonstration of significant intergroup differences by ANOVA. Differences with *p* < 0.05 were considered significant.

### Early ADME

*HPLC/UV–MS Method*. UV/LC–MS chromatographic analyses were carried out as previously reported^72^ with only few modifications. Chromatographic separations were accomplished at room temperature (rt), using a gradient elution made up of solvents A (H_2_O) and B (ACN), both acidified with 0.1% v/v formic acid (FA). The analyses began with 100% A (t = 0–1 min), dropped to 20% (t = 1–15 min), stayed at 20% (t = 15–19 min), and then returned in 1.0 min to the original conditions. The flow rate was 600 µL/min, while 10 µL was the volume injected. Spectra were acquired in both positive and negative modes within the scan range of *m/z* 100–2000, with UV detection being monitored at 254 nm.

*Plasma Stability and stability in the cellular medium.* A DMSO stock solution of tested compounds was incubated in the presence of human plasma (55.7 µg protein/mL) and HEPES buffer (25 mM, 140 mM NaCl, pH 7.4) or DMEM 10% FBS, (1% PS, and 1% Glu) at 37 °C under shaking. At selected time points (0, 0.083, 0.25, 0.5, 1, 2, 8, 24 h), samples were treated with cold ACN to stop reactions through protein denaturation, and centrifuged at 5000 rpm for 10 min. The supernatant was analyzed by UV/LC-MS to monitor the amount of unmodified compound. Data were calculated with Excel and plotted using GraphPad Prism 8.0 (GraphPad Software Inc., San Diego, CA, USA). The half-life value (t_1/2_) was calculated with the following formula: $\:{t}_{1/2}=0.693/b$

Where b is the slope found in the linear fit of the natural logarithm of the fraction remaining of the parent compound vs. incubation time.

*Microsomal stability.* In the presence of phosphate buffer (PBS 10 mM, pH 7.4), human liver microsomes (HLM) (0.2 mg mL^−1^), and a NADPH solution in MgCl_2_ 48 mM, each DMSO compound solution was incubated at 37 °C for 1 h under shaking. Then, the metabolizing reactions were stopped by adding cold acetonitrile (ACN). After centrifuging and drying the reaction mixes under N_2_ flow, the quantitative analyses were performed using the UV/LC-MS method previously described. The percentages of the metabolized and unmetabolized compounds were calculated as previously described^73,74^.

*PAMPA assay.* The DMSO stock solution of each compound was diluted 1:1 v/v with phosphate buffer (PBS 10 mM, pH 7.4) to obtain donor solutions. Each well of the filter plate was covered with a 1% w/v L-α-phosphatidylcholine solution (PC) in dodecane used to mimic the phospholipidic bilayer and then added with the donor. In the acceptor plate, a mixture solution of DMSO/PBS 1:1 v/v was placed, and the sandwich was incubated at rt for 4 h under gentle shaking. At the end time point, samples were taken from both the upper and the lower plates and analyzed using the UV/LC-MS method described above. Apparent permeability (P_app_ cm/s x 10^−6^) and membrane retention (MR %) was calculated as previously reported^75^.

## Electronic supplementary material

Below is the link to the electronic supplementary material.

Supplementary Material 1

## Data Availability

Processed data that support the findings of this study are provided within the manuscript or the supplementary information file.
