# Supplementary material for: Exploiting the bile acid binding protein as transporter of a Cholic Acid/Mirin bioconjugate for potential applications in liver cancer therapy
Source: Sci Rep. 2024 Sep 28;14:22514. doi: 10.1038/s41598-024-73636-w (PMC11439058; doi:10.1038/s41598-024-73636-w)

**SUPPORTING INFORMATION**

Exploiting the Bile Acid Binding Protein as Transporter of a Cholic Acid/Mirin Bioconjugate

for Potential Applications in Liver Cancer Therapy

Giusy Tassone^1^, Samuele Maramai^1,*^, Marco Paolino^1^, Stefania Lamponi^1^, Federica Poggialini^1^, Elena Dreassi^1^, Elena Petricci^1^, Stefano Alcaro^2,3^, Cecilia Pozzi^1,*^, Isabella Romeo^2,3^

^1^Department of Biotechnology, Chemistry and Pharmacy - University of Siena, Via Aldo Moro 2, I-53100, Siena, Italy

^2^ Department of Health Science - University “Magna Græcia” of Catanzaro, Campus “S. Venuta”, Viale Europa, 88100 Catanzaro, Italy

^3^ Net4Science Academic Spin-Off - University “Magna Græcia” of Catanzaro, Campus “S. Venuta”, Viale Europa, 88100 Catanzaro, Italy

^4^ Consorzio Interuniversitario Risonanze Magnetiche di Metallo Proteine (CIRMMP) - Via Sacconi 6, I-50019 Sesto Fiorentino (FI), Italy

* Correspondence

Dr Samuele Maramai, e-mail [samuele.maramai@unisi.it](mailto:samuele.maramai@unisi.it)

Prof Cecilia Pozzi, e-mail [cecilia.pozzi@unisi.it](mailto:cecilia.pozzi@unisi.it)

**Tables**

|  | | **BABP-DCA** | **BABP-UDCA** | **BABP-LCA** | **BABP-CDCA** | | **BABP-CA-M11** |
| --- | --- | --- | --- | --- | --- | --- | --- |
| PDB ID codes | | 9ETE | 9ETD | 9ETF | 9ETC | | 9ETG |
|  | |  | **DATA COLLECTION STATISTICS** | | | | |
| Diffraction source | I04 (DLS) | | I24 (DLS) | I04 (DLS) | I04 (DLS) | | I04 (DLS) |
| Wavelength (Å) | 0.97949 | | 0.97949 | 0.97949 | 0.95373 | | 0.97949 |
| Temperature (K) | 100 | | 100 | 100 | 100 | | 100 |
| Detector | Eiger2 XE 16M | | Eiger2 XE 16M | Eiger2 XE 16M | Eiger2 XE 16M | | Eiger2 XE 16M |
| Crystal-detector distance (nm) | 201.7 | | 258.2 | 257.8 | 231.6 | | 220.4 |
| Exposure time (s) | 0.005 | | 0.005 | 0.005 | 0.005 | | 0.008 |
| Space group | P2_1_ | | P2_1_ | P2_1_ | P2_1_ | | P 2_1_ 2_1_ 2_1_ |
| No. of subunits in ASU | 2 | | 2 | 2 | 2 | | 3 |
| a, b, c (Å) | 36.52, 60.38, 64.11 | | 36.33, 60.63, 61.67 | 36.63, 61.16, 64.04 | 36.56, 60.76, 63.55 | | 59.56, 60.57, 135.14 |
| β (°) | 99.18 | | 95.90 | 96.09 | 99.55 | | - |
| Resolution range (Å) | 63.28-2.10 (2.21-2.10) | | 61.34-2.30 (2.42-2.30) | 63.67-2.20 (2.32-2.20) | 62.67- 1.65 (1.74-1.65) | | 135.14-2.00 (2.11-2.00) |
| Total no. of reflections | 31339 (4633) | | 84269 (12742) | 42036 (5933) | 230475 (34159) | | 461786 (68740) |
| No. of unique reflections | 13482 (2018) | | 11971(1755) | 13885 (2057) | 32846 (4767) | | 33892 (4859) |
| Completeness (%) | 83.5 (85.5) | | 100.0 (100.0) | 96.4 (98.4) | 99.2 (98.6) | | 100.0 (100.0) |
| Multiplicity | 2.3 (2.3) | | 7.0 (7.3) | 3.0 (2.9) | 7.0 (7.2) | | 13.6 (14.1) |
| (I / σ(I)) | 5.1 (2.2) | | 17.7 (2.4) | 10.4 (2.2) | 16.4 (2.2) | | 14.5 (2.3) |
| R_meas_ | 0.128 (0.444) | | 0.048 (0.740) | 0.068 (0.532) | 0.049 (0.917) | | 0.096 (1.179) |
| Overall B factor from Wilson plot (Å^2^) | 26.5 | | 58.6 | 34.7 | 30.2 | | 43.0 |
|  | |  | **REFINEMENT STATISTICS** | | | | |
| Resolution range (Å) | 36.07-2.10 (2.15-2.10) | | 61.34-2.30 (2.36-2.30) | 36.45-2.20 (2.26-2.20) | 43.66-1.65 (1.69-1.65) | 67.57-2.00  (2.05-2.00) | |
| Completeness (%) | 83.22 (85.74) | | 99.99 (100.0) | 96.29 (98.41) | 99.18 (98.43) | 99.96 (100.0) | |
| No. of reflections, working set | 12801 (991) | | 11375 (812) | 13272 (1003) | 31255 (2280) | 32189 (2356) | |
| No. of reflections, test set | 679 (55) | | 584 (64) | 607 (46) | 1581 (108) | 1624 (123) | |
| Final R_cryst_ | 0.2417 (0.303) | | 0.2204 (0.351) | 0.2086 (0.279) | 0.2026 (0.373) | 0.2219 (0.295) | |
| Final R_free_ | 0.3466 (0.397) | | 0.2796 (0.351) | 0.2777 (0.329) | 0.2619 (0.366) | 0.2856 (0.346) | |
| No. of non-H atoms |  | |  |  |  |  | |
| Protein | 1896 | | 1824 | 1836 | 1923 | 2901 | |
| Ligand | 112 | | 56 | 77 | 104 | 68 | |
| Water | 236 | | 31 | 140 | 234 | 163 | |
| Total | 2244 | | 1911 | 2053 | 2261 | 3132 | |
| R.m.s. deviations bonds (Å) | 0.007 | | 0.006 | 0.007 | 0.007 | 0.010 | |
| Angles (°) | 1.518 | | 1.586 | 1.578 | 1.568 | 1.800 | |
| Average B factor (Å^2^) | 33.6 | | 65.5 | 47.7 | 38.9 | 46.9 | |
| Estimate error on coordinates based on R value (Å) | 0.421 | | 0.369 | 0.306 | 0.109 | 0.180 | |
| Ramachandran plot |  | |  |  |  |  | |
| Most favored (%) | 97.1 | | 95.5 | 97.1 | 97.6 | 98.4 | |
| Allowed (%) | 2.9 | | 4.5 | 2.9 | 2.4 | 1.6 | |

**Table S1.** Data collection and refinement statistics. Values for the outer shell are given in parentheses.

**Table S2**. Extraction Recovery % (% Rec) of CA-M11 and Mirin in human liver microsomes. Data are reported as the mean ± standard deviation (SD) of *n*=3 experiments run in triplicates.

| **Compound** | **Recovery**  **(% Rec, Time 0’)** |
| --- | --- |
| **Mirin** | 92.32 ± 1.92 |
| **CA-M11** | 69.94 ± 4.23 |

**Table S3**. PAMPA Assay. ^a^Apparent permeability (P_app_) reported in 10^-6^ cm/sec. ^b^ Percentage of membrane retention. Data are reported as the mean ± standard deviation (SD) of n=3 experiments run in triplicates.

| **Compounds** | **P_app_^a^** | **MR (%)^b^** |
| --- | --- | --- |
| **Mirin** | 0.03 ± 0.02 | 1.16 ± 0.90 |
| **CA-M11** | 0.04 ± 0.001 | 2.26 ± 0.64 |

**Figures**

**Figure S1**. 3D interaction of (**A**) DCA, (**B**) LCA, (**C**) CDCA, (**D**) UDCA in complex with cL-BABP site 2. The protein is reported as crème cartoon, with the residues involved in pivotal contacts shown as carbon sticks. The H-Bonds are interactions are indicated as yellow dashed lines.

**
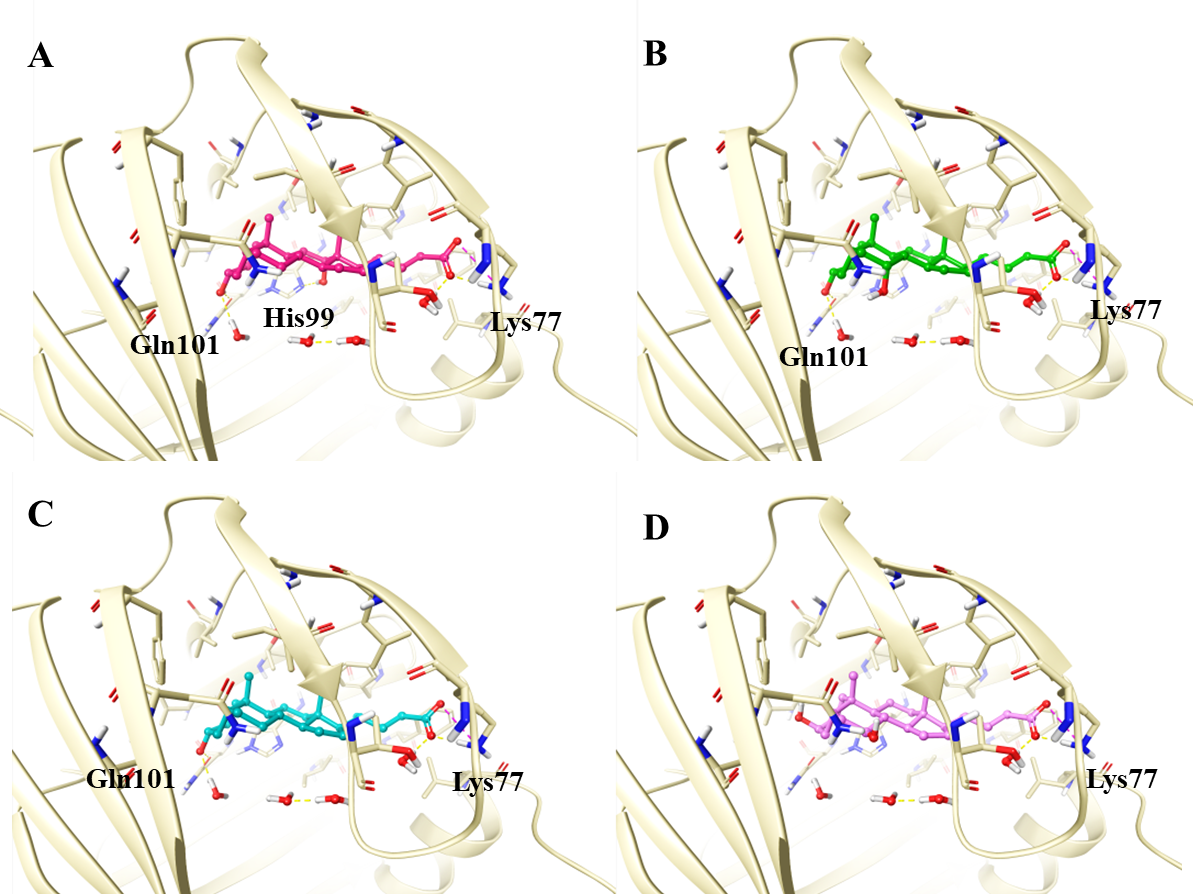
**

**Figure S2**. Relative energies and key geometry data for tautomers 1 (**6a**) and 2 (**6b**) of CA-M11 following Monte Carlo conformational searches post-processed using DFT calculations. Energies are reported as relative gas phase in M06-2X/cc-pvtz(-f) energies (GPEs) and relative solution phase (M06-2X/ cc-pvtz(-f) energies (SPEs) in water.


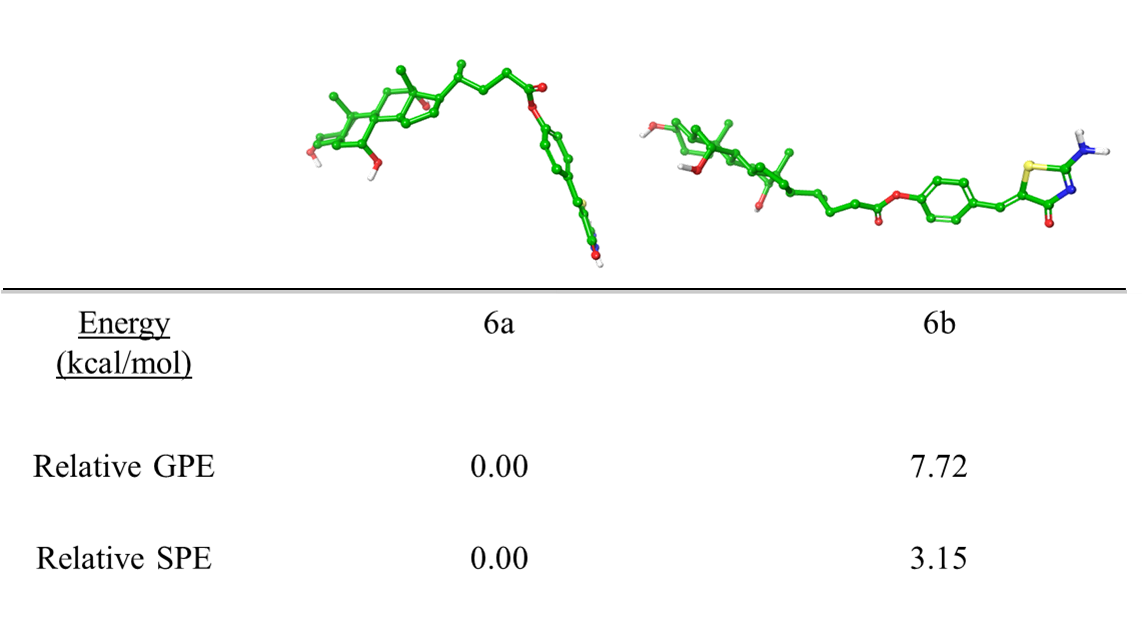


**Figure S3**. Superposition between the best docked pose of **6a** and **6b** and the bioactive pose obtained from the x-ray crystallography.

**
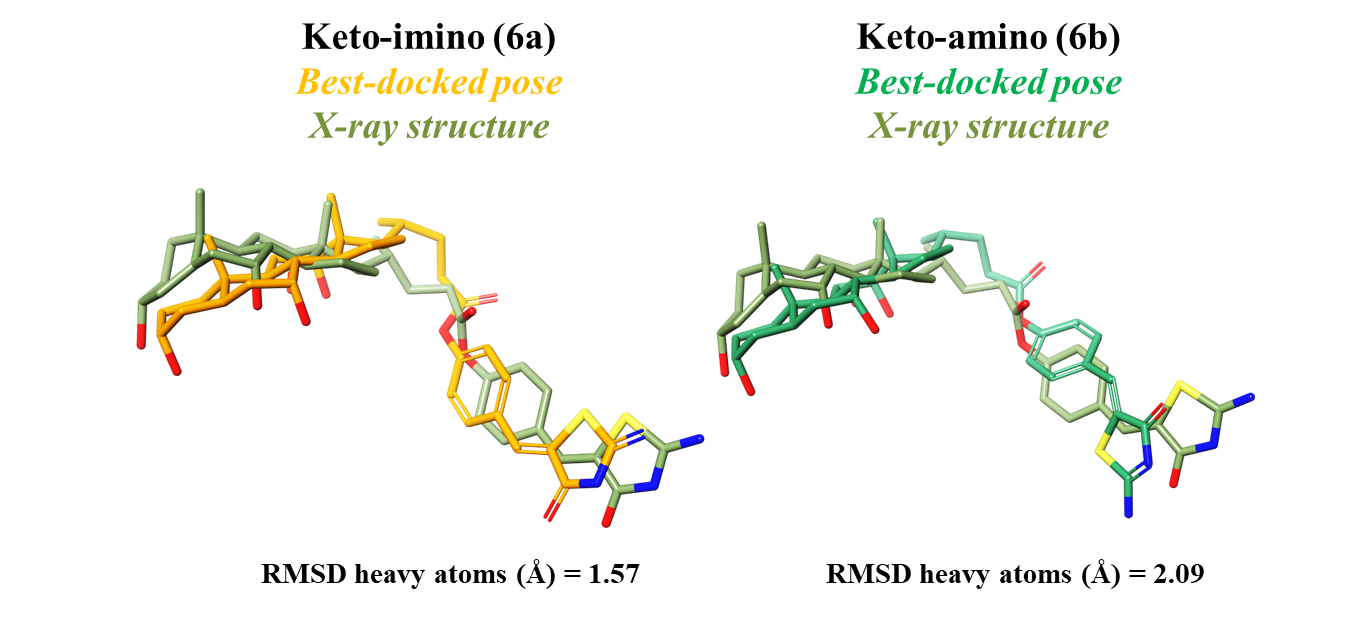
**

**Figure S4**. Ames test performed on S. typhimurium strain TA98 for A) Doxorubicin; B) Mirin; C) CA-M11; D) Doxorubicin + Mirin 5 µM; E) Doxorubicin + CA-M11 5 µM. Star ( ): Fold inc. over BL >=2.


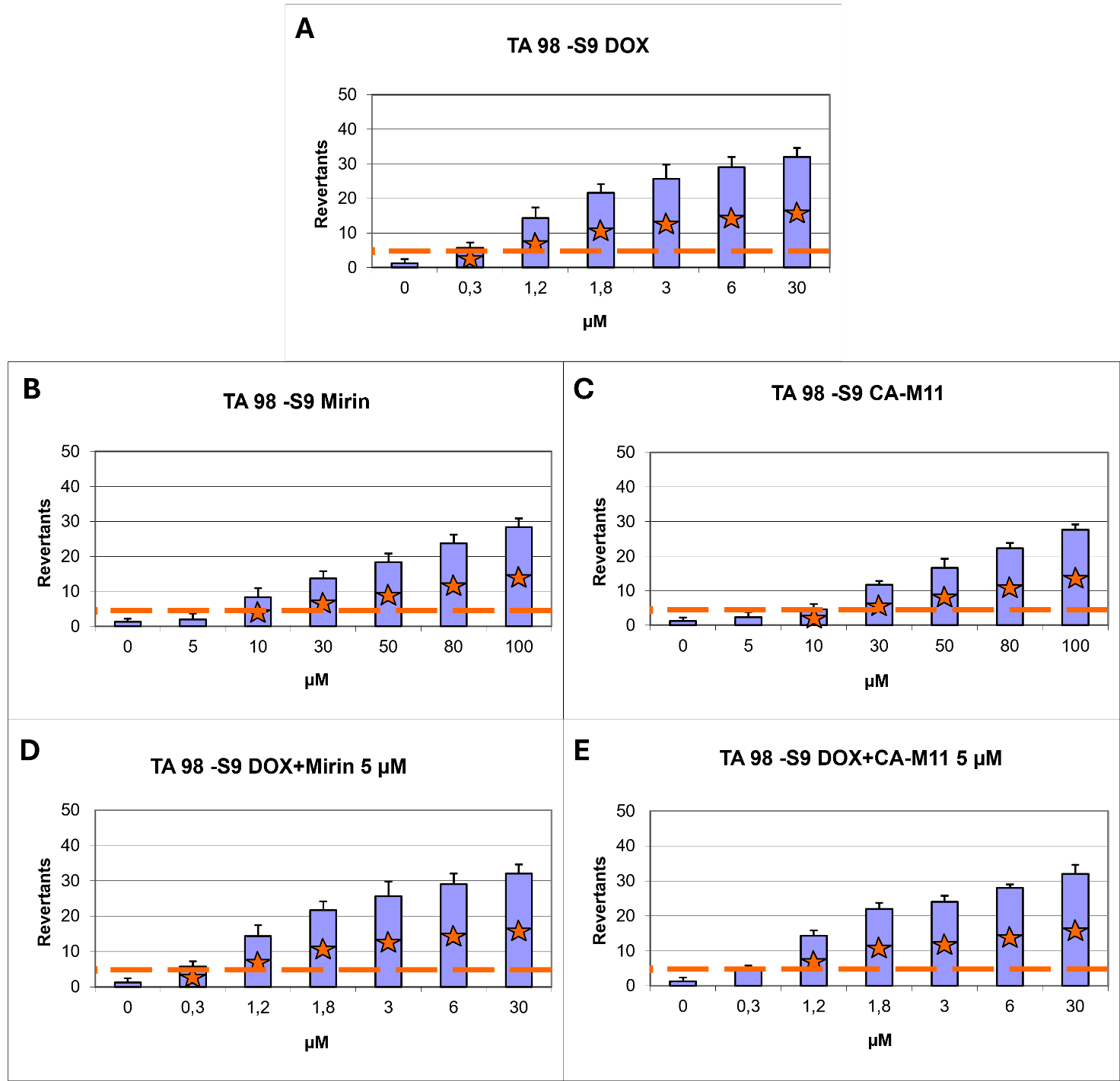


**Figure S5**. Ames test performed on S. typhimurium strain TA100 for A) Doxorubicin; B) Mirin; C) CA-M11; D) Doxorubicin + Mirin 5 µM; E) Doxorubicin + CA-M11 5 µM. Star ( ): Fold inc. over BL >=2.


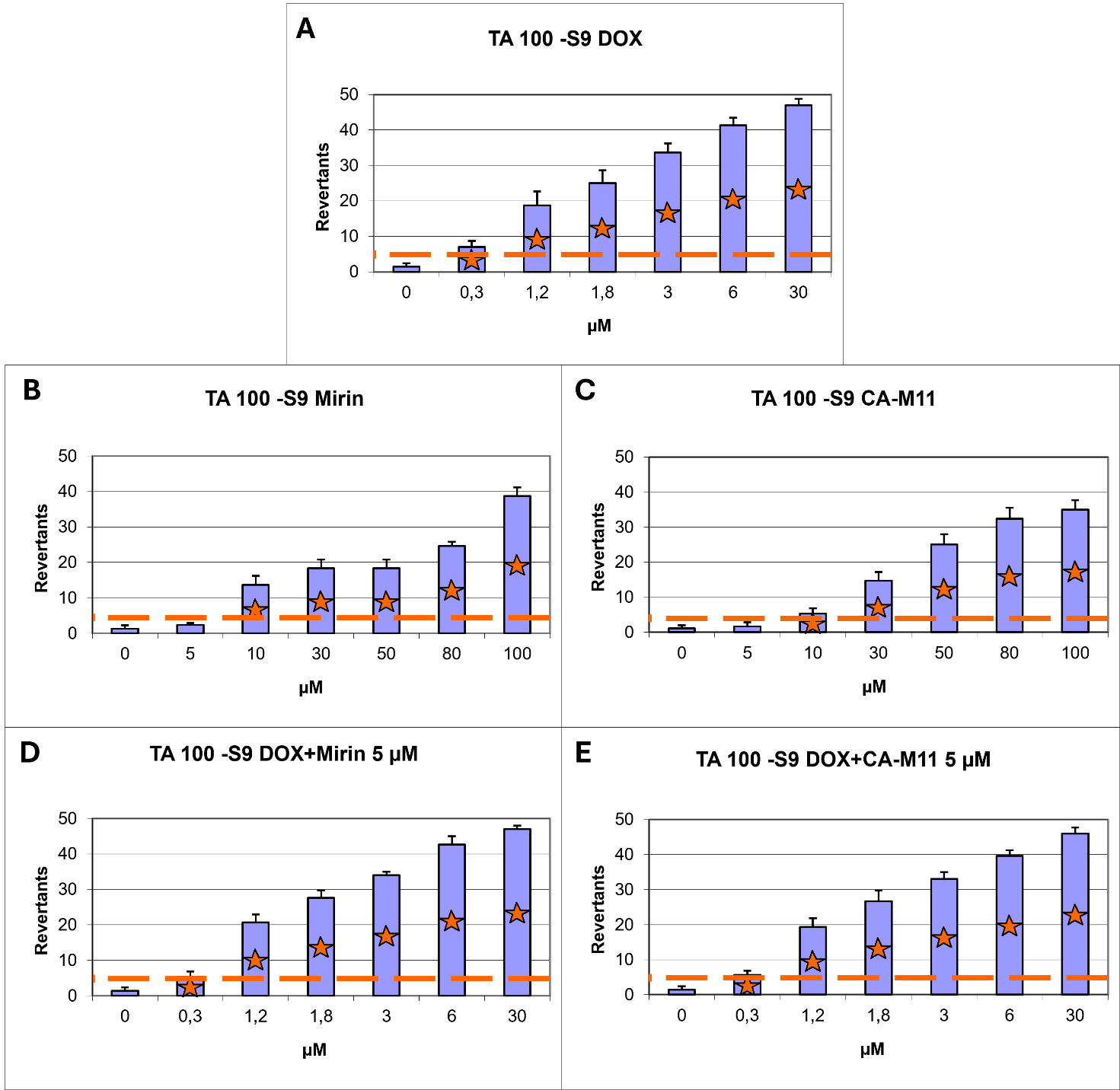


**Figure S6**. HRMS analysis report for Mirin (**5**).


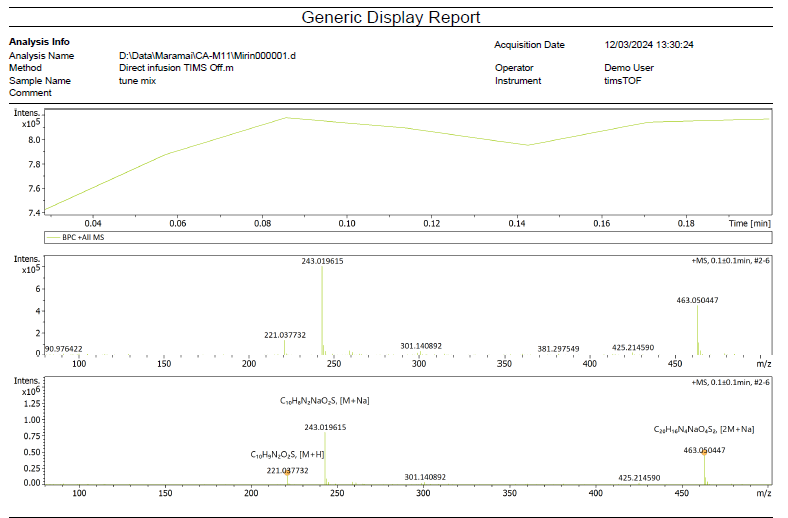


**Figure S7**. LC-MS analysis report for compound CA-M11 (**6a**)


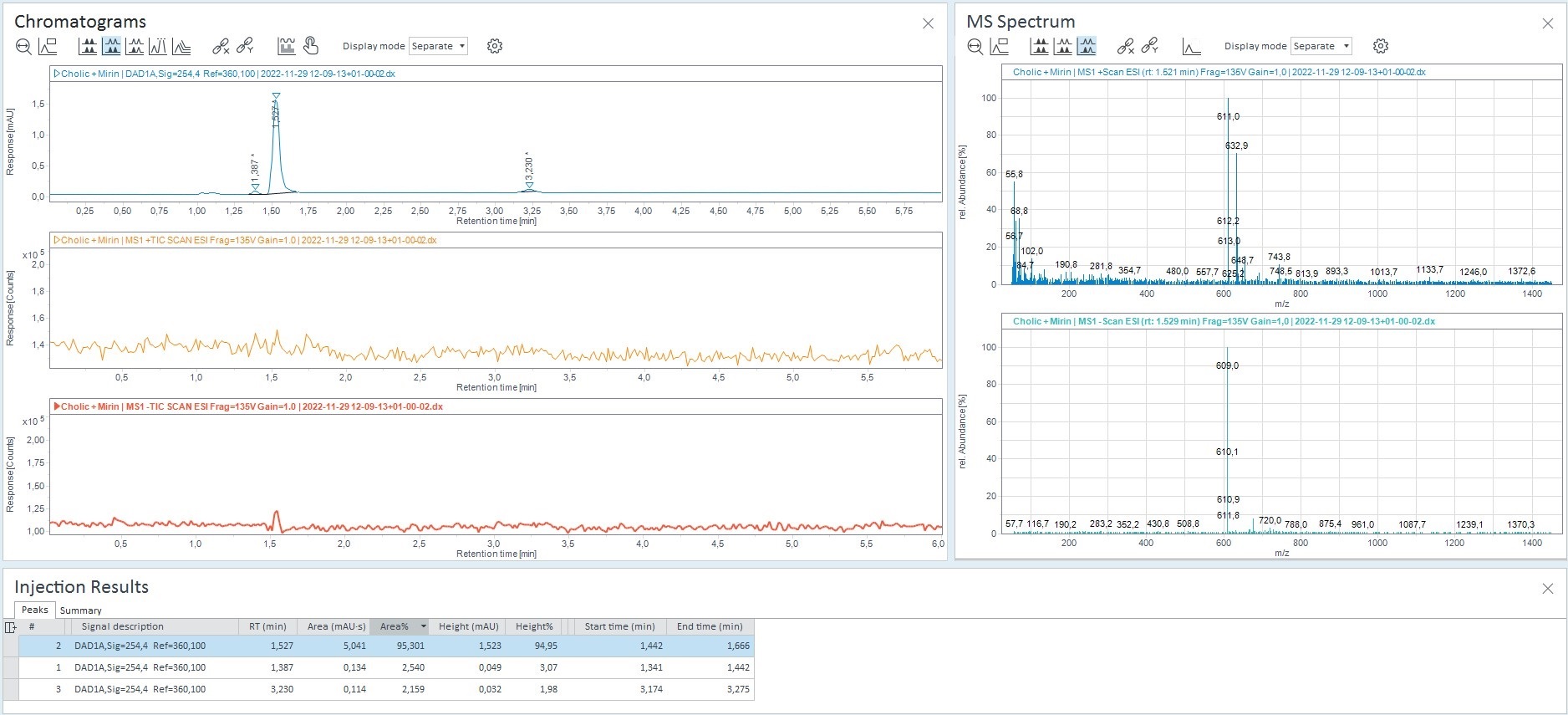


**Figure S8**. HRMS analysis report for compound CA-M11 (**6a**).


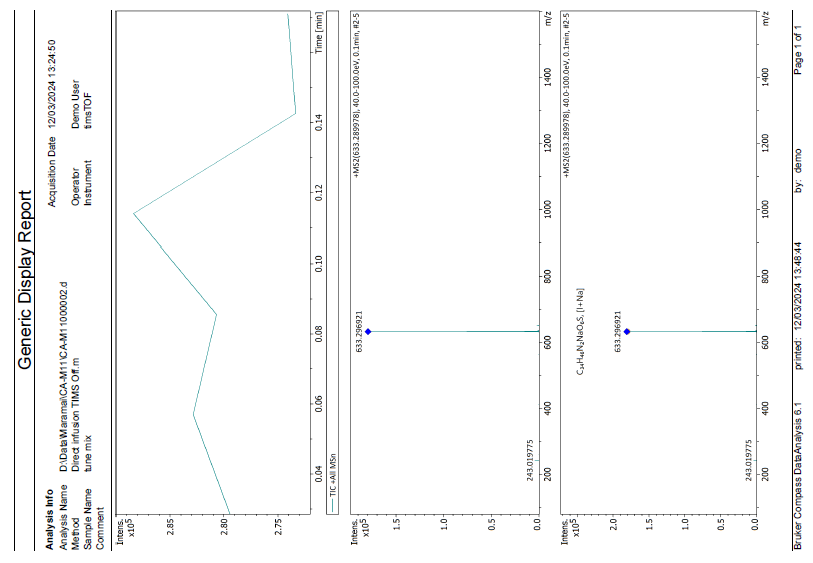

Supplement: Supplementary file 1 — Supplementary Material 1 [file 41598_2024_73636_MOESM1_ESM.docx]
